# Supplementary material for: Phylogeography of Geopsammodius Gordon and Pittino, 1992 (Coleoptera: Scarabaeidae: Aphodiinae): A radiation of endemic psammophilic beetles
Source: PLoS One. 2026 Aug 12;21(8):e0351948. doi: 10.1371/journal.pone.0351948 (PMC13466012; doi:10.1371/journal.pone.0351948)
Supplement: Supplementary file 2 [file pone.0351948.s005.pdf]

PLOS One

Phylogeography of Geopsammodius Gordon and Pittino, 1992 (Coleoptera: Scarabaeidae: Aphodiinae): a radiation of endemic psammophilic beetles

--Manuscript Draft--

**Manuscript Number:**	PONE-D-26-10786
**Article Type:**	Research Article
**Full Title:**	Phylogeography of Geopsammodius Gordon and Pittino, 1992 (Coleoptera: Scarabaeidae: Aphodiinae): a radiation of endemic psammophilic beetles
**Short Title:**	Phylogeography of Geopsammodius: a radiation of endemic psammophilic beetles
**Corresponding Author:**	Kyle E. Schnepp<br>North Carolina State University<br>Raleigh, North Carolina UNITED STATES OF AMERICA
**Keywords:**	classification; Florida; phylogeny; subterranean
**Abstract:**	Geopsammodius are sand-dwelling beetles found throughout the southeastern United States, Texas, and Honduras. Their modified morphology including reduced flight wings, reduced eyes, expanded tibiae, and fused elytra suggests their evolutionary history may be closely tied to the sand dunes in which they inhabit. Here, using six genes, we reconstruct the relationships of species within the genus and members of the tribe Psammodiini. We then performed divergence time estimation in order to determine the age of the group. We recovered Parapsammodius outside the Psammodiini indicating the need for future taxonomic revision. Within the genus Geopsammodius, we found two major clades that largely correspond with geography. The first occurring along the east coast and Gulf Coast and the second clade occurring inland in Florida and Georgia. We find that the age of Geopsammodius coincides with the formation of extensive sand dunes (20–23 MYA) suggesting that the group may have evolved concurrently with this sandy habitat.
**Order of Authors:**	Kyle E. Schnepp
	Natalie A. Saxton
	Gareth S. Powell
	Nicole L. Gunter
	Paul E. Skelley
	Ronald D. Cave
**Additional Information:**	
**Question**	**Response**
**Financial Disclosure**<br><br>Enter a financial disclosure statement that describes the sources of funding for the work included in this submission. Review the submission guidelines for detailed requirements. View published research articles from *PLOS ONE* for specific examples.<br><br>This statement is required for submission and **will appear in the published article** if the submission is accepted. Please make sure it is accurate.	The author(s) received no specific funding for this work.

**Funded studies**<br>Enter a statement with the following details:<br>• Initials of the authors who received each award<br>• Grant numbers awarded to each author<br>• The full name of each funder<br>• URL of each funder website<br>• Did the sponsors or funders play any role in the study design, data collection and analysis, decision to publish, or preparation of the manuscript?<br><br>Did you receive funding for this work?	
**Competing Interests**<br><br>Use the instructions below to enter a competing interest statement for this submission. On behalf of all authors, disclose any competing interests that could be perceived to bias this work—acknowledging all financial support and any other relevant financial or non-financial competing interests.<br><br>This statement is required for submission and **will appear in the published article** if the submission is accepted. Please make sure it is accurate and that any funding sources listed in your Funding Information later in the submission form are also declared in your Financial Disclosure statement.<br><br>View published research articles from *PLOS ONE* for specific examples.	The authors have declared that no competing interests exist.

Powered by Editorial Manager® and ProduXion Manager® from Aries Systems Corporation

**NO authors have competing interests**<br><br>Enter: *The authors have declared that no competing interests exist.*<br><br>**Authors with competing interests**<br><br>Enter competing interest details beginning with this statement:<br><br>*I have read the journal's policy and the authors of this manuscript have the following competing interests: [insert competing interests here]*<br><br>* typeset	
**Ethics Statement**<br><br>Enter an ethics statement for this submission. This statement is required if the study involved:<br><br>• Human participants<br>• Human specimens or tissue<br>• Vertebrate animals or cephalopods<br>• Vertebrate embryos or tissues<br>• Field research<br><br>Write "N/A" if the submission does not require an ethics statement.<br><br>General guidance is provided below. Consult the submission guidelines for detailed instructions. **Make sure that all information entered here is included in the Methods section of the manuscript.**	N/A

**Format for specific study types**<br><br>**Human Subject Research (involving human participants and/or tissue)**<br>• Give the name of the institutional review board or ethics committee that approved the study<br>• Include the approval number and/or a statement indicating approval of this research<br>• Indicate the form of consent obtained (written/oral) or the reason that consent was not obtained (e.g. the data were analyzed anonymously)<br><br>**Animal Research (involving vertebrate animals, embryos or tissues)**<br>• Provide the name of the Institutional Animal Care and Use Committee (IACUC) or other relevant ethics board that reviewed the study protocol, and indicate whether they approved this research or granted a formal waiver of ethical approval<br>• Include an approval number if one was obtained<br>• If the study involved *non-human primates*, add *additional details* about animal welfare and steps taken to ameliorate suffering<br>• If anesthesia, euthanasia, or any kind of animal sacrifice is part of the study, include briefly which substances and/or methods were applied<br><br>**Field Research**<br><br>Include the following details if this study involves the collection of plant, animal, or other materials from a natural setting:<br>• Field permit number<br>• Name of the institution or relevant body that granted permission	
**Data Availability**<br><br>Authors are required to make all data underlying the findings described fully available, without restriction, and from the time of publication. PLOS allows rare exceptions to address legal and ethical concerns. See the PLOS Data Policy and FAQ for detailed information.	Yes - all data are fully available without restriction

A Data Availability Statement describing where the data can be found is required at submission. Your answers to this question constitute the Data Availability Statement and **will be published in the article**, if accepted.<br><br>**Important:** Stating 'data available on request from the author' is not sufficient. If your data are only available upon request, select 'No' for the first question and explain your exceptional situation in the text box.<br><br>Do the authors confirm that all data underlying the findings described in their manuscript are fully available without restriction?	
**Describe where the data may be found in full sentences. If you are copying our sample text, replace any instances of XXX with the appropriate details.**<br><br>• If the data are **held or will be held in a public repository**, include URLs, accession numbers or DOIs. If this information will only be available after acceptance, indicate this by ticking the box below. For example: *All XXX files are available from the XXX database (accession number(s) XXX, XXX.).*<br>• If the data are all contained **within the manuscript and/or Supporting Information files**, enter the following: *All relevant data are within the manuscript and its Supporting Information files.*<br>• If neither of these applies but you are able to provide **details of access elsewhere**, with or without limitations, please do so. For example:<br><br>*Data cannot be shared publicly because of [XXX]. Data are available from the XXX Institutional Data Access / Ethics Committee (contact via XXX) for researchers who meet the criteria for access to confidential data.*<br><br>*The data underlying the results presented in the study are available from (include the name of the third party*	All sequences will be available via NCBI (https://www.ncbi.nlm.nih.gov/genbank/), they are being processed and will be available before publication.

and contact information or URL).<br>• This text is appropriate if the data are owned by a third party and authors do not have permission to share the data.<br><br>* typeset	
Additional data availability information:	Tick here if the URLs/accession numbers/DOIs will be available only after acceptance of the manuscript for publication so that we can ensure their inclusion before publication.

Powered by Editorial Manager® and ProduXion Manager® from Aries Systems Corporation

Manuscript Click here to access/download;Manuscript;Manuscript.docx

Phylogeography of *Geopsammodius* Gordon and Pittino, 1992 (Coleoptera: Scarabaeidae: Aphodiinae): a radiation of endemic psammophilic beetles

Kyle E. Schnepp[*,1,2,3], Natalie A. Saxton[4], Gareth S. Powell[3], Nicole L. Gunter[5], Paul E. Skelley[1], Ronald D. Cave[6]

[1]Florida State Collection of Arthropods, Division of Plant Industry, Florida Department of Agriculture and Consumer Services, Gainesville, Florida, USA

[2]Entomology and Nematology Department, University of Florida, Gainesville, Florida, USA

[3]Department of Entomology and Plant Pathology, North Carolina State University, Raleigh, North Carolina, USA

[4]Department of Biology, Brigham Young University, Provo, Utah, USA

[5]Biodiversity and Geosciences Program, Queensland Museum, South Brisbane, Queensland, Australia

[6]Indian River Research and Education Center, Entomology and Nematology Department, Ft. Pierce, Florida, USA

* Corresponding author

E-mail: keschnepp@gmail.com (KS)

Abstract

Geopsammodius are sand-dwelling beetles found throughout the southeastern United States, Texas, and Honduras. Their modified morphology including reduced flight wings, reduced eyes, expanded tibiae, and fused elytra suggests their evolutionary history may be closely tied to the sand dunes in which they inhabit. Here, using six genes, we reconstruct the relationships of species within the genus and members of the tribe Psammodiini. We then performed divergence time estimation in order to determine the age of the group. We recovered *Parapsammodius* outside the Psammodiini indicating the need for future taxonomic revision. Within the genus *Geopsammodius,* we found two major clades that largely correspond with geography. The first occurring along the east coast and Gulf Coast and the second clade occurring inland in Florida and Georgia. We find that the age of *Geopsammodius* coincides with the formation of extensive sand dunes (20–23 MYA) suggesting that the group may have evolved concurrently with this sandy habitat.

Keywords: classification; Florida; phylogeny; subterranean

Introduction

A number of traditionally surface-dwelling terrestrial lineages have shifted to underground habitats, be it to shelter from weather or predators, to find food, or to nest in. Throughout the animal kingdom, subterranean animals with special modifications to live underground include some amphibians, fish, insects, lizards, mammals, marsupials, millipedes, and spiders [1–6]. These modifications can include enlarged and shortened legs, long and thin elongated legs, addition or loss of hair or setae, reduction or loss of vision, and loss of pigment [7]. The combination of subterranean habitats and the often limited dispersal abilities of these animals allow for the investigation of unique selective pressures on the evolutionary history of these taxa [8]. The field of subterranean biology includes many different microhabitat types, including caves, rock fissures, scree slopes, alluvial and colluvial material, and seepage springs, as well as the microclimates that subterranean habitats experience [9–16]. While subterranean habitats such as larger caves allow for humans to physically enter and search for insects, this habitat also often requires specialized collecting techniques to properly survey. This field of study is growing, and many recent publications discuss habitat types and the specialized methods needed to study these inaccessible environments [17– 22].

Subterranean animals have disparate levels of adaptation and requirements for living underground. The ability to fly is one of the major evolutionary adaptations that allowed insects to become the dominant and most diverse form of life on earth. The loss of flight is significant as it limits dispersal and finding mates, food, shelter, and proper substrate for laying eggs [23]. In spite of the many costs of going flightless or moving underground, occupying subterranean microhabitats might represent an adaptive zone for many lineages as there are underutilized resources, less competition, and protection from predators. Subterranean species are often associated with homogeneous environments, such as deserts, islands, and caves, and can sometimes reach rather large population sizes in their specific niche [24].

There are many examples of insects moving underground and developing adaptations for subterranean life, including species of Blattodea, Coleoptera, Diptera, Hemiptera, Hymenoptera, Orthoptera, and Thysanura [25–32]. Among the major groups of insects, Coleoptera, or beetles, make up a significant portion of the subterranean fauna. Beetles may be especially well suited to a subterranean lifestyle as their generally more heavily sclerotized cuticle allows for easier digging and can take more wear from abrasion, and the hardened elytra protect the body from compression.

Within beetles, several families, including Coccinellidae, Dryopidae, Dytiscidae, Elmidae, Histeridae, Hydrophilidae, Noteridae, Tenebrionidae, and Zopheridae, have at least a few members specializing in underground habitats [33–41]. Lineages in Salpingidae [42] and Dermestidae [43] have also specialized to live in anthropogenic situations to such a degree that they are essentially subterranean. Other families (i.e., Carabidae, Curculionidae, Leiodidae, and Staphylinidae) have a large number of disparately related species that occur in subterranean habitats and have significant adaptations for this specialized environment [44–52].

The scarab subfamily Aphodiinae is composed of 12 generally accepted tribes, 386 genera, and approximately 3,600 species and occurs worldwide [53]. Several Old World tribes are not confidently placed within the subfamily, indicating that the broader limits of Aphodiinae are not yet settled (MJ Paulsen, personal communication). Future effort to expand the available molecular data and increase the taxon sampling will likely elucidate more accurate relationships. One common name for the Aphodiinae is “small dung beetles” or “aphodiine dung beetle”, distinguishing them from the Scarabaeinae, or “dung beetles”. While these common names may be accurate in that many genera and species are obligately associated with various animal dung, there are many large lineages that appear to have no association with dung at all, occupying decaying detritus, tree holes, and various nests. Even within the dung feeding clades, there are many atypical behaviors and niche preferences that show extreme specialization. One niche that is commonly colonized is in association with accumulations of sand, where the

beetles likely consume tiny pieces of organic matter. Many tribes have a large number of taxa that are specialized and isolated to different sandy microhabitats, called psammophiles. Some of these psammophiles occur in massive dunes systems that span hundreds of square kilometers, while others are associated with alluvial or colluvial systems that are made of silt, clay, or gravel but also contain a portion of sand (Figs 1A–C). One tribe of Aphodiinae, the Psammodiini, are almost exclusively associated with sandy habitats.

Fig 1. Habitats where *Geopsammodius* are found and size comparison of an individual *Geopsammodius* with sand grains. (A) Archbold Biological Station, Lake Placid, Florida. (B) Scrub habitat near Naples, Florida. (C) Ohoopee Dunes near Reidsville, Georgia. (D) *Geopsammodius hydropicus* (Horn).

Placement of genera into Psammodiini is generally done with the following suite of characters: head distinctly granulate; pronotum with up to five transverse furrows separated by swellings; longitudinal furrow often present on the pronotum; elytra with a basal bead; pygidium with a long basal groove; and metatarsomeres often triangularly expanded, especially the basal tarsomere. Additional diagnostic features include the type of male genitalia and details of the epipharyngeal and maxillary structures of the mouthparts [54]. The tribe Psammodiini contains approximately 26 genera and 400 species worldwide, with 15 genera and 89 species occurring in the New World. When looking at the Psammodiini in relation to other Aphodiinae, especially Eupariini, problems with the classification arise. While the diagnostic characters given above are useful, they are not explicitly synapomorphies. Cartwright [55} discusses the similarities between *Psammodius* Fallén, currently split into several different genera, and *Ataenius* Harold and suggests that members of both genera should probably be placed in the same tribe of Aphodiinae. Most, if not all, Psammodiini are associated with sand or sandy soils, and it is

expected that the morphology of members in this niche would reflect this. Adaptations typical of subterranean beetles (within a substrate) include reduced eyes and pigmentation, shortened legs, loss of flight wings, and fusion of the elytra. Many of the diagnostic characters for the tribe may simply be homoplasious, convergent within specific clades of Aphodiinae, due to their similar lifestyle.

Within the Psammodiini, the genus *Geopsammodius* ~~Gordon and Pittino~~ was proposed for two species previously in *Psammodius*, *Psammodius hydropicus* Horn (the type species for the new genus) and *Psammodius relictillus* Deyrup and Woodruff [56]. A third species was described by Lavalette [57], and an additional eight species were described by Skelley [58], who provided natural history notes and a key to species and proposed species-groups based on morphological characters. These groups consist of the relictillus species-group with five species, the hydropicus species-group with two species, and the rileyi species-group with two species. *Geopsammodius atlantida* Skelley and *Geopsammodius sabinae* Lavalette were ungrouped. He also made note of difficulties in understanding the distribution and species delimitation of *Geopsammodius rileyi* Skelley and *Geopsammodius unsidensis* Skelley in the coastal and inland sand dunes of Texas.

Species of *Geopsammodius* differ from those in the genus *Psammodius* and most of the Psammodiini in having significantly reduced eyes and flight wings (Figs 1D, 2A). With reduced eyes and reduced dispersal capabilities, populations are inclined to become isolated, and there is significant potential for speciation events. As is presently understood, species in the genus inhabit current and ancient sand dunes in the southeastern United States (Fig 3) and Honduras, where they are found in loose, soft sand. Dunes began to form in peninsular Florida approximately 23 MYA [59] as sand was moved to the region and deposited, and they have been growing at various times since, depending on sea level. These active and relictual dunes contain many isolated and endemic species that occur nowhere else [60–65]. A proposed sister genus, *Leiopsammodius* Rakovic, has similar natural history and morphological characters but

has fully functional eyes and wings. Due to the lack of additional characters, it is unclear whether reduced eyes and wings are a synapomorphy shared by a monophyletic *Geopsammodius* or if this character state belongs to a lineage nested in the middle of *Leiopsammodius*. Here, we test the monophyly of *Geopsammodius* and the Psammodiini and, using fossil calibration and divergence time estimation, compare the clade age of *Geopsammodius* with relevant geologic events of the region.

Fig 2. Dorsal habitus of Psammodiini. (A) *Geopsammodius relictillus* (Deyrup and Woodruff). (B) *Leiopsammodius deyrupi* Harpootlian, Gordon, and Woodruff. (C) *Neopsammodius quinqueplicatus* (Horn). (D) *Platytomus longulus* (Cartwright).

Fig 3. Map showing the distribution of *Geopsammodius* across the southeastern USA.

Materials and methods

Taxon sampling

Taxon sampling included both freshly collected field samples and existing museum specimens to cover all extant described species in the region. In total, 15 specimens representing nine of the 11 species were included in the phylogenetic analyses, including all Nearctic taxa (S1 Table). vouchers are deposited in the Florida State Collection of Arthropods (FSCA), Gainesville, FL, USA, with the exception of *Ataenius alternatus* (Melsheimer) that is deposited in the Queensland Museum, AUS. Nine specimens from recent sampling done by the first author were used as outgroups, comprising seven genera.

Molecular data

DNA extractions were completed using Qaigen's DNeasy blood and tissue kit (Qiagen, CA, USA). Due to their small body size, non-destructive DNA extractions were performed on whole specimens by soaking the entire specimen in lysis buffer for 24 hours. The rest of the standard manufacturer protocol was followed except for the last elution step which was repeated twice with only half the suggested volume used each time. All genes, except for those associated with *A. alternatus,* were completed using polymerase chain reactions (PCR) with the primers and conditions listed in Tables 1 and 2. Six genes were targeted including three nuclear, 18S (~780 bps), 28S (~490 bps), and ITS2 (~440 bps), and three mitochondrial, 16S (~485 bps), COI (~640 bps), and COII (~750 bps). These sequences are available via GenBank and genus, species, locality, and unique identifiers can be found in S1 Table. Successful PCR products were sequenced at FDACS-DPI Molecular Diagnostics Laboratory, Gainesville, FL, USA, DNA Sequencing Center.

Table 1. Gene regions, primer names, and primer sequences used in this study.

Region	Primer	Sequence
16S	16SF	5' CGC CTG TTT ATC AAA AAC AT 3'
16S	16SR	5' CTC GGT TTG AAT CAG ATC A 3'
18S	18SH17F	5' AAA TTA CCC ACT CCC GGC A 3'
18S	18SH35R	5' TGG TGA GGT TTC CCG TGT T 3'
28S	D2F	5' CGG GTT GCT TGA GAG TGC AGC 3'
28S	D2R	5' GGT CCG TGT TTC AAG ACG GG 3'
COI	LCO 1490	5' GGT CAA CAA ATC ATA AAG ATA TTG G 3'
COI	HCO 2198	5' TAA ACT TCA GGG TGA CCA AAA AAT CA 3'
COII	F-lue	5' TCT ATT ATG GCA GAT TAG TGC 3'
COII	R-lys	5' GAG ACC AGT ACT TGC TTT CAG TCA TC 3'
ITS2	ITS2F	5' TGT GAA CTG CAG GAC ACA TG 3'
ITS2	ITS2R2	5' TCT CGC CTG CTC TGA GGT 3'

Table 2. Primers and PCR conditions used in this study to amplify selected gene regions.

Primers	Thermocycle Protocols
16SF/16SR	1) 95C/2 min; 33x steps 2–4: 2) 98C/30 sec; 3) 50C/30 sec; 4) 72C/40 sec; 5) 72C/7 min; 6) 4C/∞
18SH17F/18SH35R	1) 95C/2 min; 35x steps 2–4: 2) 98C/30 sec; 3) 52C/45 sec; 4) 72C/1 min; 5) 72C/7 min; 6) 4C/∞
D2F/D2R	1) 95C/2 min; 35x steps 2–4: 2) 98C/30 sec; 3) 60C/30 sec; 4) 72C/1 min; 5) 72C/7 min; 6) 4C/∞
LCO/HCO	1) 95C/2 min; 33x steps 2–4: 2) 98C/30 sec; 3) 50C/30 sec; 4) 72C/2 min; 5) 72C/7 min; 6) 4C/∞
F-lue/R-lys	1) 95C/2 min; 33x steps 2–4: 2) 95C/1 min; 3) 51C/1 min; 4) 72C/ 2 min; 5) 72C/10 min; 6) 4C/∞
ITS2F/ITS2R2	1) 95C/2 min; 33x steps 2–4: 2) 98C/30 sec; 3) 56C/30 sec; 4) 72C/40 sec; 5) 72C/7 min; 6) 4C/∞

Genes for *A. alternatus* were extracted from Ultraconserved Element data that was generated using the Scarab_3kv1 probe set [66]. Reads were cleaned using Illumiprocessor [67], and were assembled using SPADES [68]. Target genes (see paragraph above) were searched for and extracted by using the “Map to reference” function in Geneious (Biomatters, https://www.geneious.com) with *Ataenius* spp. (Genbank accessions: GU226586, EU156751, EF487637) and *Aphodius* (MW412426) used as the reference. Resulting sequences for both PCR and UCE generated data were examined for quality, trimmed, and aligned in Geneious v2022.2.2 [69] using MAFFT v.7 [70] before being concatenated for subsequent analyses (total aligned length, 3,836bps).

Phylogenetic reconstruction

Tree reconstruction was performed under maximum likelihood using IQ-TREE 2 [71] and ModelFinder [72]. Due to the rapidly evolving nature of the non-coding region ITS2 as compared to coding genes, trees both with and without the region were run. The resulting topology with ITS2 was used as a fixed topology to estimate divergence times in subsequent analyses. To

use this topology, the relative ages (or heights) of nodes that were going to be constrained based on fossil evidence were adjusted by using the function *chronopl* applied in the R program Ape [73] so that their relative ages fell within the prior distributions of our constraints.

Divergence time estimation and lineage through time plot

BEAUti was used to prepare an xml file for subsequent analysis in BEAST v.2.7.6 [74]. The site model was set based on the results of ModelFinder (i.e., GTR+F+I+G4). We used a partitioned clock model such that a strict mitochondrial clock rate was applied to all mitochondrial genes (i.e., 0.0115) by using the standard rate found in insects [75]. The use of this mitochondrial clock was employed in conjunction with a fossil prior to estimate divergence times. We placed an exponential fossil prior (hard minimum 35 MYA, soft maximum 67.5 MYA) on the node for the MRCA of *Ataenius* based on the fossil *Ataenius damzeni* Bukejs and Alekseev, 2018 from Baltic amber. The soft maximum was based on the oldest estimated age of Eocene aphodiine fossils [76], with the 95% quantile placed at 56 MYA. Two older Cretaceous fossils were not included due to the difficulty of placing ambiguous compression fossils. Divergence time estimation was completed in BEAST. We ran multiple combinations of tree (i.e., Birth-Death and Yule) and clock models on nuclear genes (Relaxed Clock Log Normal and Relaxed Clock Exponential) with a chain length of 10 or 30 million. Results were analyzed in Tracer v.1.7.2 to ensure convergence. A consensus tree was generated for each tree and clock model combination with a 10% burn-in using TreeAnnotator. Final topologies were visualized in FigTree v.1.4.4 [77] and Adobe Illustrator v.29.0.1. The tree used for the final dated phylogeny included ITS2. A lineage through time plot was generated from the results of our divergence time estimation (Yule, Relaxed Clock Exponential) by using the “ltt.plot” function in *phytools* [78].

Results

Topology

The tribe Psammodiini was recovered as non-monophyletic with respect to the included Eupariini outgroups. *Parapsammodius*, traditionally treated in the Psammodiini, was found within a clade including the diverse genus *Ataenius* sister to the remaining Psammodiini. The two species of *Leiopsammodius* (Fig 2B) were recovered sister to *Platytomus* (Fig 2D). The clade *Leiopsammodius*+*Platytomus* was recovered as a sister group to the remaining clade of *Neopsammodius* plus a monophyletic *Geopsammodius*. The species of *Geopsammodius* formed two major groups with high support. Within these two major groups, four clades were recovered with variable support. Three of these clades loosely agree with previously described informal species-groups [58], but some notable differences warrant further investigation.

Due to the nature of noise within a non-coding region such as ITS2 at higher levels, analyses were run with (S1 Fig) and without (S2 Fig) this region included. In both phylogenetic analyses, the topology was mostly stable, and most relationships maintained high support. However, two problematic taxa with low support were present. *Geopsammodius morrisi* Skelley and *Geopsammodius ohoopee* Skelley consistently had low support (i.e., <50% BS) in their placement in all trees. Additionally, *G*. *ohoopee* changed position between the two major clades recovered in this analysis.

Clade ages

Divergence time estimation (Fig 4) recovered similar clade ages across all tree and clock models, with the Birth-Death model recovering slightly older ages (Table 3). The clade age for Psammodiini+Eupariini was recovered as 69–82 MYA. With the exclusion of *Parapsammodius*, a monophyletic Psammodiini was recovered with a clade age of 63–75 MYA. The recovered age for *Geopsammodius* is estimated to be 19–24 MYA, with most extant species appearing 7–11 MYA (Fig 4). The age recovered for the related genus *Leiopsammodius* is around 43–47

MYA. The lineage through time plot recovered a value of 0.033 for the slope of the line representing the average accumulation of lineages over time. Visual inspection of the time plot shows a sharp increase in lineage accumulation after 20 MYA, corresponding to the estimated age of the *Geopsommadius* clade as well as the proposed timing of sand dune formations in Florida (20–23 MYA [59]).

Fig 4. Results of divergence time estimation (Yule, Relaxed Clock Exponential) for *Geopsammodius* and related outgroups. Bootstrap values <95% are shown at nodes. Vertical red bar indicates the estimated age for the early formation of most sand dunes in the southeastern United States. Lineage through time plot shows a steep increase in the number of lineages immediately following early sand dune formation.

Table 3. Results of all clock and tree models. Mean ages are in millions of years and the 95% HPD in brackets below. MRCA = most recent common ancestor.

Tree Model	Clock Model	MRCA *Geopsammodius*	MRCA non-monophyletic "Psammodiini"
Yule	Relaxed clock log normal	19.40 [13.81–25.72]	69.56 [51.95–91.13]
Yule	Relaxed clock exponential	19.34 [14.23–25.80]	69.32 [51.67–90.50]
Birth-death	Relaxed clock log normal	24.02 [17.28–31.97]	82.48 [60.46–107.78]
Birth-death	Relaxed clock exponential	23.19 [16.27–30.90]	79.97 [57.22–105.59]

Discussion

Systematics

From the phylogenetic results, it appears that *Geopsammodius* evolved from a common ancestor (i.e., monophyletic) likely similar to *Neopsammodius*. *Neopsammodius* (Fig 2C) is a genus that is widespread in North and Central America, and its species have normally developed eyes and functional flight wings. The current distribution of *Geopsammodius* (Fig 3) is largely restricted to the Gulf Coast, and its species have reduced eyes and vestigial flight wings.

It appears that *Parapsammodius* is more closely related to *Ataenius* and may belong in the Eupariini, if the Eupariini are separate from the Psammodiini. Characters used to include *Parapsammodius* in Psammodiini are the coarsely granulate head, dentate clypeus, and vertex lacking swellings, but these characters may not always be informative in the Aphodiinae. In the description and diagnosis for *Parapsammodius* [54], the authors state that, while some external characters lean toward Psammodiini, the mouthparts are of the Eupariini type. Examination of additional taxa are required to understand whether or not Psammodiini and Eupariini are largely monophyletic, if the tribes contain individual lineages that need to be reclassified, or if the two tribes need to be combined.

Here, we used the fossil *Ataenius damzeni* for divergence time estimation. A second fossil, *Ataenius europaeus* [79] also found in Baltic amber, has also been described. The description of *A*. *europaeus* certainly indicates it belongs in the Aphodiinae and most likely Eupariini, but comparison of the species with the current concept of *Ataenius* is impossible. Bukejs & Alekseev [80] supported the placement of *A*. *europaeus* in *Ataenius*, with 13 morphological characters. This example highlights the problematic nature of the genus as it is currently defined. While many of these characters allow for exclusion of a defined genus or tribe, no character or suite of characters define present-day *Ataenius*. Many species previously

placed in *Ataenius* have been moved based on specific, unique characters, thus leaving *Ataenius* as a dumping ground for potentially disparately related species. Additional analyses with much higher taxon sampling than has been done to date is necessary to create a well-supported phylogeny to test the monophyly of not only *Ataenius* but also Eupariini and Psammodiini.

We were able to include nine of the eleven species from the genus *Geopsammodius* in our analyses. One species that was not able to be included was *G. sabinae* from French Guiana. Before specimens were available, Skelley [58] remarked on possible similarities of this species to *Geopsammodius atlantida* Skelley. After seeing specimens, however, it is clear that *G*. *sabinae* does not belong in *Geopsammodius* and is most likely a member of *Odontopsammodius* without clypeal teeth. The inclusion of *G*. *atlantida* in this study was not possible due to lack of fresh material. Additional populations or species of *Geopsammodius* are likely to occur between the known populations of southern Texas and the one in Honduras. It is also possible that more species or populations occur farther south in Central America or even in the West Indies. In addition to *Geopsammodius*, there are many additional subterranean beetles, in sandy soils in Florida, that have been described [81–85], but much additional work in this niche is necessary to clarify relationships and understand the true diversity of the region.

Additional surveying is needed in North and Central America, as specimens are rarely collected without employing specialized collecting methods. Material from many of the Texas populations would allow for additional study of the populations and determine if current species concepts and distributions are accurate. It would also elucidate whether additional species exist or one widespread variable species occurs in Texas as well as how *G*. *atlantida* is related to the rest of *Geopsammodius*. Similarly, additional material would allow for a study of the relictillus-group in Florida and whether clades seen in the current phylogeny represent speciation or they are simply an artifact of the distribution in the taxon sampling. Fresh specimens of untested populations of *Geopsammodius* and genera (i.e., *Odontopsammodius*, *Psammodius*,

Rhyssemus) are necessary to get an accurate understanding of the tribe and how Psammodiini and Eupariini are related. A thorough evaluation of distributions and relationships cannot be inferred without additional specimens.

Biogeography

While the current phylogeny maintained high support throughout most of the tree, additional genes could clarify certain relationships as well as more definitely place *G*. *morrisi* and *G*. *ohoopee,* both of which had lower nodal support in our phylogenies. This would allow for a more accurate interpretation of the evolutionary history of *Geopsammodius* and provide insight into additional geologic events that may have influenced ancient speciation events.

At present, the two major clades in *Geopsammodius* appear to broadly follow a geologic origin. One clade, *hydropicus*+*rileyi*+*subpedalis*+*unsidensis*+*withlacoochee*, occurs along the east coast and Gulf Coast. The second clade, *fuscus*+*morrisi*+*ohoopee*+*relictillus*, occurs inland in Florida and Georgia. Within these groups, *G*. *hydropicus* (current east coast) is sister to *G*. *subpedalis* (current Florida panhandle coast), and *hydropicus*+*subpedalis* is sister to *G*. *withlacoochee*. This relationship would indicate that, at some point, *G*. *withlacoochee* may have been separated during a glacial maximum allowing *hydropicus*+*subpedalis* to maintain itself until it, too, was separated during a recent glacial retreat. The Texas *rileyi*+*unsidensis* lineage is sister to the coastal Florida clade, indicating that at some point an ancestor of the coastal Florida clade may have dispersed past the Mississippi River, likely moving with barrier sand islands. After this potential dispersal event, the lineage split into *G*. *rileyi* and *G*. *unsidensis*, one mainly coastal and one mainly inland. This hypothesis is complicated, however, by remaining confusion over distribution and species limits. The position of *G*. *ohoopee* is also problematic in trees with and without ITS2. There is low support for its placement in both trees, and the species changed position between the two major clades in each tree. *Geopsammodius ohoopee*

is also unusual in that it appears to be restricted to riverine and wind deposited sand dunes in Georgia, unlike the remaining species that are associated with marine deposits.

The second inland clade, without *G*. *ohoopee*, is divided into two clades, one of these being composed of “*relictillus*”, or near, and the other comprising *“fuscus+morrisi”*. The *G*. *relictillus* portion of the tree indicates significant divergence and potentially new species as the two representatives from the Lake Wales Ridge are distinct from specimens collected in areas west of the ridge. Additional specimens and study of material might answer how different these populations may be. The placement of *G*. *morrisi* is also problematic as it has low support in both trees. The distribution of *G*. *morrisi* is also unusual in that it occurs in an area just east of, and is not distinctly differentiated from, the Lake Wales Ridge. Despite lower support, the phylogeny indicates that *G*. *morrisi* is sister to the two *G*. *fuscus* populations included in the study. *Geopsammodius fuscus* has been collected near the coast, but in sand deposits distinctly separate from the current dune system and intracoastal waterways. This would suggest that a population east of the Lake Wales Ridge, separate from *G*. *relictillus*, was established and speciated as sea levels rose and fell.

Geologic events that may have created new habitats and niches for animals in the southeastern USA are significant in number. A series of events, specifically in relation to the dated phylogeny, appear especially relevant. For most of the Cretaceous period (~100 MYA) through the Eocene (34 MYA), the Gulf Trough (Suwannee Straight) was a connection between the Gulf of Mexico and Atlantic Ocean (Fig 5). This trough and the current running from southwest to northeast, as well as higher sea levels during this period, allowed for a continued separation of the continental alluvial deposits and the carbonate deposits of the shallow sea covering modern day Florida [59, 86–87]. In the late Oligocene and early Miocene (23 MYA), sea levels dropped, and the Appalachian Mountains experienced uplift, which in turn increased erosion rates. This decrease in sea level and increase in erosion filled the Gulf Trough and connected what had been two different depositional environments in the panhandle and

peninsular Florida. This connection allowed for massive amounts of siliciclastic deposits to be moved south through longshore currents, covering much of the carbonate platform that had formed over the prior 100 million years.

Fig 5. The Georgia Seaway in the present day southeastern USA filled in approximately 23 MYA. As the Seaway filled, eroded sands reached carbonate rocks deposited in a shallow sea over present-day Florida. [Redrawn from Hine 2013]

Once present over Florida, these sand deposits were moved and shaped through hundreds or thousands of cycles of sea level rise and fall, shifting between 100 feet higher than today to 400 feet lower. These cycles created, destroyed, and moved islands and dune systems, thus creating many opportunities for speciation events, especially for animals with poor dispersal abilities [88]. The development of this new habitat and niche 20–23 MYA corresponds to the origin of *Geopsammodius* 19–24 MYA. The crown age of *Geopsammodius* coinciding with the beginning of the sand dune formation in Florida, and the subsequent speciation and radiation of this genus aligning with the continued deposition of sand across this region, suggests the coevolution of *Geopsammodius* with this sandy habitat. Once specialized for a subterranean niche by losing flight wings and having extremely reduced eyes, this lineage would have lower dispersal capabilities. Individuals would generally be subject to moving with sand, although it is possible that gene flow could occur with some specimens being blown, washed, or rafted to suitable habitat.

While testing this very close association between the origin of these beetles and the time of sand dune formation sheds light on the evolutionary history of this group, it is also likely an association shared by additional taxa of the region. Sand dunes, specifically in peninsular Florida, are home to a variety of endemic plant species, including Avon Park rattlebox (*Crotalaria avonensis* K.R. DeLaney and Wunderlin), scrub balm (*Dicerandra frutescens*

Shinners), scrub eryngo (*Eryngium cuneifolium* Small), scrub spurge (*Euphorbia rosescens* E.L. Bridges and Orzell), and Florida ziziphus (*Pseudoziziphus celata* (Judd and D.W. Hall) Hauenschild) [60, 65]. Several endemic animals that are only found in these dunes include the Florida scrub jay (*Aphelocoma coerulescens* (Bosc)), the Florida sand skink (*Plestiodon reynoldsi* (Stejneger)), and the Florida scrub lizard (*Sceloporus woodi* Stejneger) [89–90]. In addition to these animals, there are many Coleoptera restricted to the loose, deep sands of the Lake Wales Ridge of central Florida, such as *Anomala eximia* Potts, *Cicindelidia highlandensis* (Choate), *Enaphalodes archboldi* Lingafelter and Chemsak, *Mycterus marmoratus* Pollock, *Odontotaenius floridanus* Schuster, *Onychomira floridensis* Campbell, *Phyllophaga okeechobea* Robinson, *Phyllophaga panorpa* Sanderson, *Pleotomodes needhami* Green, *Plesioclytus relictus* Giesbert, *Romulus globosus* Knull, *Selonodon archboldi* Galley, and *Serica frosti* Dawson [61–64, 91–92]. These beetle species are relatively recently described, with the year of description averaging to 1976, very late compared to most other eastern North American species. This implies how rarely collected most of these species are and indicates that most of them inhabit very restricted and isolated niches. Such specific associations with a unique microhabitat like sand dunes leads to increased vulnerability these taxa face related to the conservation of those habitats. It is well documented that sand dune systems, especially those in Florida, are facing several risks (i.e., land use, natural disasters, sea level rise) [93–94]. Consequently, those risks are shared by the closely associated taxa that inhabit the dune systems. Studies like the present one help identify those associations and the taxa most at risk.

Acknowledgments

For assistance in molecular sequencing we thank the Molecular Diagnostics Laboratory, Gainesville, FL, USA, Matthew Moore, and Cheryl Roberts. We thank Kate Love for making maps, Mayumi Needham for assistance with figures, and Brandon Woo for additional fresh

specimens. We thank Akito Kawahara, Jiri Hulcr, and Rebecca Kimball for their insights and guidance with this project. We also thank the Florida Department of Agriculture and Consumer Services, Division of Plant Industry, for support of this work.

Author contributions

Conceptualization: Kyle E. Schnepp, Gareth S. Powell.

Data curation: Kyle E. Schnepp, Natalie A. Saxton.

Formal analysis: Kyle E. Schnepp, Natalie A. Saxton.

Methodology: Kyle E. Schnepp, Natalie A. Saxton, Gareth S. Powell.

Validation: Nicole L. Gunter, Paul E. Skelley, Ronald D. Cave.

Writing – original draft preparation: Kyle E. Schnepp, Natalie A. Saxton, Gareth S. Powell.

Writing – review & editing: Kyle E. Schnepp, Natalie A. Saxton, Gareth S. Powell, Nicole L. Gunter, Paul E. Skelley, Ronald D. Cave.

Funding

The authors received no specific funding for this work.

Competing interests

The authors have declare that no competing interests exist.

Data availability statement

Sequence data used in this study are available via GenBank. Genus, species, locality, and unique identifiers can be found in S1 Table.

References

1. Hillis DM, Chamberlain DA, Wilcox TP, Chippindale PT. A new species of subterranean blind salamander (Plethodontidae: Hemidactyliini: *Eurycea*: *Typhlomolge*) from Austin, Texas, and a systematic revision of central Texas paedomorphic salamanders. Herpetologica 2001; 57:266–280.

2. Kliukin NS, Nguyen TV, Le SX, Bragin AM, Tran TTV, Gorin VA, et al. A new species of the genus *Dibamus* Duméril & Bibron, 1839 (Squamata: Dibamidae) from the driest and hottest place of Vietnam. Zootaxa 2023; 5380:301–320. https://doi.org/10.11646/zootaxa.5380.4.1

3. Marek PE, Buzatto BA, Shear WA, Mean JC, Black DG, Harvey MS, et al. The first true millipede—1306 legs long. Scientific Reports 2021; 11:1–8.

4. Ohara WM, Costa ID Da, Fonseca ML. Behaviour, feeding habits and ecology of the blind catfish *Phreatobius sanguijuela* (Ostariophysi: Siluriformes). Journal of Fish Biology 2016;89:1285–1301. https://doi.org/10.1111/jfb.13037

5. Sifuentes-Romero IS, Aviles AM, Carter JL, Chan-Pong A, Clarke A, Crotty P, et al. Trait loss in evolution: What cavefish have taught us about mechanisms underlying eye regression. Integrative and Comparative Biology 2023; 63:393–406. https://doi.org/10.1093/icb/icad032

6. Springer MS, Emerling CA, Gatesy J. Three blind moles: Molecular evolutionary insights on the tempo and mode of convergent eye degeneration in *Notoryctes typhlops* (southern marsupial mole) and two chrysochlorids (golden moles). Genes 2023; 14:1–33. https://doi.org/10.3390/genes14112018

7. Deharveng L, Bedos A. Diversity of terrestrial invertebrates in subterranean habitats. In: Moldovan O, Kováč Ľ, Halse S (eds.), Cave Ecology. Ecological Studies vol *235*. 2018:107–172.

8. Mammola S, Amorim IR, Baguette ME, Vorges PAV, Cheeptham N, Cooper SJB, et al. Fundamental research questions in subterranean biology. Biological Reviews 2020; 95:1855–1872. https://doi.org/10.1111/brv.12642

9. Badino G. Underground meteorology - "What's the weather underground?". Acta Carsologica 2010; 39:427–448. https://doi.org/10.3986/ac.v39i3.74

10. Culver DC, Pipan T. Superficial subterranean habitats - gateway to the subterranean realm? Cave and Karst Science 2008 [2009]; 35:5–12.

11. Culver DC, Pipan. The Biology of Caves and Other Subterranean Habitats. Oxford, UK: Oxford University Press, 2009.

12. Culver DC, Holsinger JR, Feller DJ. The fauna of seepage springs and other shallow subterranean habitats in the Mid-Atlantic Piedmont and Coastal Plain. Northeastern Naturalist 2012; 19:1–42. https://doi.org/10.1656/045.019.m901

13. Eusébio RP, Fonseca PE, Rebelo R, Luz Mathias M da, Reboleira ASPS. How to map potential mesovoid shallow substratum (MSS) habitats? A case study in colluvial MSS. Subterranean Biology 2023; 45:141–156. https://doi.org/10.3897/subtbiol.45.96332

14. Nitzu E, Nae A, Giurginca A, Popa I. Invertebrate communities from the mesovoid shallow substratum of the Carpatho-Euxinic area: Eco-faunistic and zoogeographic analysis. Travaux de l'Institut de Spéologie "É. Racovitza" 2010; 49:41–79.

15. Nitzu E, Nae A, Băncilă R, Popa I, Giurginca A, Plăiaşu R. Scree habitats: ecological function, species conservation and spatial-temporal variation in the arthropod community. Systematics and Biodiversity 2014; 12:65–75. https://doi.org/10.1080/14772000.2013.878766

16. Pipan T, Culver DC. Chapter 107 Shallow subterranean habitats. In: White WB, Culver DC, Pipan T (eds.), Encyclopedia of Caves. London, UK: Academic Press, 2019; 896–908.

17. Giachino PM, Vailati D.The Subterranean Environment: Hypogean Life, Concepts and Collecting Techniques. Verona: WBA Handbooks, 2010.

18. Halse SA, Pearson GB. Troglofauna in the vadose zone: comparison of scraping and trapping results and sampling adequacy. *Subterranean Biology* 2014; 13:17–34. https://doi.org/10.3897/subtbiol.13.6991

19. López H, Oromí P. A pitfall trap for sampling the mesovoid shallow substratum (MSS) fauna. Speleobiology Notes 2010; 2:7–11.

20. Mammola S, Piano E, Giachino PM, Isaia M. An ecological survey of the invertebrate community at the epigean/hypogean interface. Subterranean Biology 2017; 24:27–52. https://doi.org/10.3897/subtbiol.24.21585

21. Owen JA. A pitfall trap for repetitive sampling of hypogean arthropod faunas. The Entomologist's Record and Journal of Variation 1995; 107:225–228.

22. Sims I, Cole J, Verdon P. Hypogean pitfall trapping: A novel technique for assessing soil biodiversity in agroecosystems. British Journal of Entomology and Natural History 2016; 29:211–229.

23. Scholtz CH. Evolution of flightlessness in Scarabaeoidea (Insecta, Coleoptera). Deutsche Entomologische Zeitschrift 2000; 47:5–28.

24. Deyrup M, Woodruff RE. A new flightless *Psammodius* from Florida's inland dunes (Coleoptera: Scarabaeidae). The Coleopterists Bulletin 1991; 45:75–80.

25. Andersen T, Baranov V, Hagenlund LK, Ivković M, Kvifte GM, Pavlek M. Blind flight? A new troglobiotic orthoclad (Diptera, Chironomidae) from the Lukina Jama-Trojama cave in Croatia. PLoS ONE 2016; 1:e0152884: 1–15. https://doi.org/10.1371/journal.pone.0152884

26. Espinasa L, Smith GB. "*Nicoletia*" *tergata* Mills, 1940 rediscovered in Florida and confirmed as the first species of the Coletiniinae (Zygentoma: Nicoletiidae) in North America. Zootaxa 2023; 5228:337–350. https://doi.org/10.11646/zootaxa.5228.3.6

27. Hlaváč P, Perreau M, Čeplík D. The Subterranean Beetles of the Balkan Peninsula. Czech University of Life Sciences Prague, Faculty of Forestry and Wood Sciences, 2017.

28. Hoch H. The Hawaiian cave planthoppers (Homoptera: Fulgoroidea: Cixiidae) - A model for rapid subterranean speciation? International Journal of Speleology 1999; 26:21–31.

29. Hubbell TH. A monographic revision of the genus *Ceuthophilus*. University of Florida Publications, Biological Sciences Series II, 1936.

30. Hubbell TH. A blind cricket-locust (*Typhloceuthophilus floridanus* n. gen. et sp.) inhabiting *Geomys* burrows in peninsular Florida (Orthoptera, Gryllacrididae, Rhaphidophorinae). Annals of the Entomological Society of America 1940; 33:10–32.

31. Mackerras MJ. A blind cockroach from the caves in the Nullarbor Plain (Blattodea: Blattellidae). Australian Journal of Entomology 1967; 6:39–44.

32. Schmidt FA, Solar RR. Hypogaeic pitfall traps: methodological advances and remarks to improve the sampling of a hidden ant fauna. Insectes Sociaux 2010; 57:261–266. https://doi.org/10.1007/s00040-010-0078-1

33. Aalbu R, Kanda K, Smith AD. Reinstatement of Eschatoporiini Blaisdell, 1906, a unique tribe of blind cavernicolous Tenebrionidae from California, with a new species from Napa County (Coleoptera, Tenebrionidae, Lagriinae). ZooKeys 2017; 688:135–149. https://doi.org/10.3897/zookeys.688.13575

34. Barr CB, Spangler PJ. A new genus and species of stygobiontic dryopid beetle, *Stygoparnus comalensis* (Coleoptera: Dryopidae), from Comal Springs, Texas. Proceedings of the Biological Society of Washington 1992; 105:40–54.

35. Bowles DE, Barr CB, Stanford R. Habitat and phenology of the endangered riffle beetle *Heterelmis comalensis* and a coexisting species, *Microcylloepus pusillus*, (Coleoptera: Elmidae)

at Comal Springs, Texas, USA. Archiv für Hydrobiologie 2003; 156:361–383. https://doi.org/10.1127/0003-9136/2003/0156-0361

36. Caterino MS, Harden C. Unseeing and unseen: On the distribution, morphology, and larva of one of North America's rarest histerid beetles, *Geocolus caecus* Wenzel (Coleoptera: Histeridae). The Coleopterists Bulletin 2022; 76:191–205. https://doi.org/10.1649/0010-065X-76.2.191

37. Ivie MA, Ślipiński SA. A new species of *Lyreus* Aubé from Alabama, first report of the genus from the New World (Zopheridae: Colydiinae: Synchitini). The Coleopterists Bulletin 2001; 55:501–505.

38. Leys R, Watts C, Cooper S, Humphreys W. Evolution of subterranean diving beetles (Coleoptera: Dytiscidae: Hydroporini, Bidessini) in the arid zone of Australia. Evolution 2003; 57:2819–2834. https://doi.org/10.1554/03-165

39. Ślipiński A, Tomaszewska KW. *Carinodulinka baja*, new genus and new species of Carinodulini from Baja California (Coleoptera: Coccinellidae). Annales Zoologici 2002; 52:489–492.

40. Spangler PJ. A new water beetle, *Troglochares ashmolei*, n. gen., n. sp., from Ecuador; the first known eyeless cavernicolous hydrophilid beetle (Coleoptera: Hydrophilidae). Proceedings of the Entomological Society of Washington 1981; 83:316–323.

41. Spangler PJ. Four new stygobiontic beetles (Coleoptera: Dytiscidae; Noteridae; Elmidae). Insecta Mundi 1996; 10:241–259.

42. Cooter J, Vávra J. *Anommatus reitteri* Ganglbauer, 1899 (Coleoptera: Bothrideridae) new to the British fauna. Entomologist's Monthly Magazine 2021; 157:173–180.

43. Zhou Y, Nicholls JA, Liu Z, Hartley D, Szito A, Ślipiński A, et al. Molecular phylogeny of Dermestidae (Coleoptera) reveals the polyphyletic nature of *Trogoderma* Latreille and the taxonomic placement of the Khapra beetle *Trogoderma granarium* Everts. Insect Systematics and Diversity 2022; 6:1–24. https://doi.org/10.1093/isd/ixac026

44. Anderson RS. A taxonomic revision of the genus *Lymantes* Schonherr, 1838 (Coleoptera: Curculionidae: Molytinae: Lymantini) in the USA. The Coleopterists Bulletin 2016; 70:111–124. https://doi.org/10.1649/072.070.0115

45. Bello C, Colonnelli E, Forbicioni L, Osella G, Ruzzier E. A new genus and species of anophthalmous Otiorhynchini from Greece, with a new synonymy and new combinations (Coleoptera: Curculionidae, Entiminae). Zootaxa 2021; 4938:69–84. https://doi.org/10.11646/zootaxa.4938.1.3

46. Delić T, Lohaj R, Brestovanský J, Čáha D, Jalžić B. Questioning the monophyly of Anthroherponina (Coleoptera: Leiodidae: Cholevinae: Leptodirini) and description of three new, ecologically ultraspecialized subterranean species. Zoological Journal of the Linnean Society 2024; 200:736–754. https://doi.org/10.1093/zoolinnean/zlad090

47. Escalona HE, Oberprieler RG. *Undarobius*, a new genus of cavernicolous weevils (Curculionidae: Entiminae: Leptopiini) from the Undara Lava Caves in north-eastern Australia,

with an overview of anophthalmic and microphthalmic Australian Curculionidae. Zootaxa 2021; 5023:207–222. https://doi.org/10.11646/zootaxa.5023.2.2

48. Grebennikov VV, Newton AF. Minute larvae of Leptotyphlinae (Coleoptera: Staphylinidae): description of three genera with discussion on the monophyly and phylogenetic position of the subfamily as inferred from larval morphology. Zootaxa 2008; 1817:49–58. https://doi.org/10.11646/zootaxa.1817.1.5

49. Hlaváč P, Oromi P, Bordoni A. Catalogue of troglobitic Staphylinidae (Pselaphinae excluded) of the world. Subterranean Biology 2006; 4:19–28.

50. Hlaváč P, Ozimec R, Pavićević D. Catalogue of the troglobitic Pselaphinae (Coleoptera, Staphylinidae) of the Balkan Peninsula, with a key to genera. In: Pavićević D, Perreau M (eds.), *Advances in the studies of the fauna of the Balkan Peninsula.* Papers dedicated to the memory of Guido Nonveiller. Monograph 22. Belgrade: Institute for Nature Conservation of Serbia, 2008:307–328.

51. Reddell JR, Cokendolpher JC. New species and records of cavernicole *Rhadine* (Coleoptera: Carabidae) from Camp Bullis, Texas. Texas Memorial Museum, Speleological Monographs 2004; 4:153–162.

52. Sugaya K, Yamasako J. A new species of the genus *Ishikawatrechus* (Coleoptera, Trechinae) from Japan. Zootaxa 2014; 3768:189–195. https://doi.org/10.11646/zootaxa.3768.2.6

53. Schoolmeester P. World Scarabaeidae Database. Catalogue of Life Checklist. 2023. Available from: https://www.catalogueoflife.org/data/taxon/8RYQ6

54. Verdú JR, Stebnicka ZT, Galante E. A new Neotropical genus of the Eupariini-Psammodiini complex with comparative morphology of mouthparts structures and analysis of characters among related taxa (Coleoptera: Scarabaeidae: Aphodiinae). Acta zoologica cracoviensia 2006; 49:55–72.

55. Cartwright OL. Scarab beetles of the genus *Psammodius* in the Western Hemisphere. Proceedings of the United States National Museum 1955; 104:413–462.

56. Gordon RD, Pittino R. Current status of the American genera and species of Psammodiini (Coleoptera: Scarabaeidae: Aphodiinae). The Coleopterists Bulletin 1992; 46:260–273.

57. Lavalette F. A new species of the genus *Geopsammodius* from French Guiana (Coleoptera: Aphodiidae: Psammodiini). Folia Heyrovskyana 1999; 7:289–292.

58. Skelley PE. A revision of the genus *Geopsammodius* Gordon and Pittino, 1992 (Scarabaeidae: Aphodiinae: Psammodiini). Insecta Mundi 2006; 20:101–112.

59. Hine AC. Geologic History of Florida. Major Events that Formed the Sunshine State. Gainesville: University Press of Florida, 2013.

60. Christman SP, Judd WS. Notes on plants endemic to Florida scrub. Florida Scientist 1990; 53:52–73.

61. Deyrup M. Arthropods endemic to Florida scrub. Biological Sciences 1989; 4:254–270.

62. Deyrup M. Arthropod footprints in the sands of time. Florida Entomologist 1990; 73:529–538.

63. Deyrup M, Carrel J. Conservation Status and Management of Lake Wales Ridge Arthropods Restricted to Scrub Habitat. Final Report on Project T-15-D: Lake Wales Ridge Scrub Arthropods to the Florida Fish and Wildlife Conservation Commission. 2011.

64. Deyrup M, Franz R. Rare and endangered biota of Florida, Volume IV. Invertebrates. Gainesville: University Press of Florida, 1994.

65. Estill JC, Cruzan MB. Phytogeography of rare plant species endemic to the southeastern United States. Castanea 2001; 66:3–23.

66. Gustafson GT, Glynn RD, Short AE, Tarasov S, Gunter NL. To design, or not to design? Comparison of beetle ultraconserved element probe set utility based on phylogenetic distance, breadth, and method of probe design. *Insect Systematics and Diversity* 2023; 7:1–16. https://doi.org/10.1093/isd/ixad014

67. Faircloth BC. illumiprocessor: a trimmomatic wrapper for parallel adapter and quality trimming. 2013. http://dx.doi.org/10.6079/J9ILL.

68. Prjibelski A, Antipov D, Meleshko D, Lapidus A, Korobeynikov A. Using SPAdes de novo assembler. Current Protocols in Bioinformatics 2020; 70:e102.

69. Kearse M, Moir R, Wilson A, Stones-Havas S, Cheung M, Sturrock S, et al. Geneious Basic: an integrated and extendable desktop software platform for the organization and analysis of sequence data. Bioinformatics 2012; 28:1647–1649.

70. Katoh K, Standley DM. MAFFT multiple sequence alignment software version 7: improvements in performance and usability. Molecular Biology and Evolution 2013; 30:772–780.

71. Minh BQ, Schmidt HA, Chernomor O, Schrempft D, Woodhams MD, Haeseler A von, et al. IQ-TREE 2: New models and efficient methods for phylogenetic inference in the genomic era. Molecular Biology and Evolution 2020; 37: 1530–1534.

72. Kalyaanamoorthy S, Minh BQ, Wong TKF, Haeseler A, Jermiin LS. ModelFinder: fast model selection for accurate phylogenetic estimates. Nature Methods 2017; 14:587–589.

73. Paradis E, Schliep K. Ape 5.0: an environment for modern phylogenetics and evolutionary analyses in R. Bioinformatics 2019; 35:526–528.

74. Drummond AJ, Suchard MA, Xie D, Rambaut A. Bayesian phylogenetics with BEAUti and the BEAST 1.7. Molecular Biology and Evolution 2012; 29:1969–1973.

75. Brower AV. Rapid morphological radiation and convergence among races of the butterfly *Heliconius erato* inferred from patterns of mitochondrial DNA evolution. Proceedings of the National Academy of Sciences 1994; 91:6491–6495. https://doi.org/10.1073/pnas.91.14.6491

76. Krell FT. Catalogue of fossil Scarabaeoidea (Coleoptera: Polyphaga) of the Mesozoic and Tertiary, Version 2007. Denver Museum of Nature and Science Technical Report 2007; 2007-8:1–79.

77. Rambaut A, Drummond AJ. FigTree Version 1.4.4. 2012. (Downloaded May 2023).

78. Revell LJ. phytools: an R package for phylogenetic comparative biology (and other things). Methods in Ecology and Evolution 2012; 2:217-223.

79. Quiel G. Bemerkungen über Coleopteren aus dem baltischen Bernstein. Berliner Entomologische Zeitschrift 1910; 55:181–192.

80. Bukejs A, Alekseev VI. A new extinct species of *Ataenius* Harold from Baltic amber (Coleoptera: Scarabaeidae: Aphodiinae). Zootaxa 2018; 4442:153–160. https://doi.org/10.11646/zootaxa.4442.1.8

81. Bousquet Y. Description of a new species of *Clivina* Latreille from southeastern United States with a key to North American species of the *fossor* group (Coleoptera: Carabidae: Clivinini). The Coleopterists Bulletin 1997; 51:343–349.

82. Bousquet Y, Skelley PE. Description of a new species of *Scarites* Fabricius (Coleoptera: Carabidae) from Florida. The Coleopterists Bulletin 2010; 64:45–49. https://doi.org/10.1649/0010-065X-64.1.45

83. Bousquet Y, Skelley PE. Description of two new species of *Clivina* Latreille (Coleoptera, Carabidae, Clivinini) from southeastern United States. ZooKeys 2012; 178:43–50. https://doi.org/10.3897/zookeys.178.2817

84. Sokolov IM, Carlton CE, Cornell JF. Review of *Anillinus* with descriptions of 17 new species and a key to soil and litter species (Coleoptera: Trechinae: Bembidiini). The Coleopterists Bulletin 2004; 58:185–233.

85. Sokolov IM, Schnepp KE. A new subterranean species of *Anillinus* Casey (Carabidae, Trechinae, Anillini) from Florida. Subterranean Biology 2021; 39:33–44. https://doi.org/10.3897/subtbiol.39.65769

86. Hine AC, Suthard B, Locker SD, Cunningham KJ, Duncan DS, Evans M, et al. Karst sub-basins and their relation to the transport of Tertiary siliciclastic sediments on the Florida Platform. In: Swart PK, Eberli GP, McKenzie JA (eds.), Perspectives in sedimentary geology: A tribute to the career of Robert N. Ginsburg. Wiley-Blackwell, 2009:179–197.

87. Lane E. Florida's Geologic History and Geologic Resources. Florida Geological Survey Special Publication 35, 1994.

88. Whittaker RJ, Triantis KA, Ladle RJ. A general dynamic theory of oceanic island biogeography. Journal of Biogeography 2008; 35:977–994. https://doi.org/10.1111/j.1365-2699.2008.01892.x

89. Mohler PE. Rare and endangered biota of Florida, Volume III. Amphibians and Reptiles. Gainesville: University Press of Florida, 1992.

90. Rodgers Jr JA, Kale II HW, Smith HT. Rare and endangered biota of Florida, Volume V. Birds. Gainesville: University Press of Florida, 1996.

91. Vlasak J. Notes on the natural history of *Enaphalodes archboldi* Lingafelter and Chemsak, 2002 and *E. bingkirki* Lingafelter and Santos-Silva, 2018 (Coleoptera: Cerambycidae). Insecta Mundi 2018; 0671:1–8.

92. Vlasak J. Observations on the natural history of *Romulus globosus* Knull (Coleoptera: Cerambycidae). Insecta Mundi 2019; 0711:1–5.

93. Brown AC, McLachlan A. Sandy shore ecosystems and the threats facing them: some predictions for the year 2025*. Environmental Conservation* 2002; 29:62–77.

94. Cox J, Kautz R, MacLaughlin M, Gilbert T. Closing the gaps in Florida's wildlife habitat conservation system. Florida Game and Fresh Water Fish Commission, Tallahassee, FL, 1994.

Supporting Information

S1 Fig. Maximum likelihood cladogram showing bootstrap support including all six genes used in this study.

S2 Fig. Maximum likelihood cladogram showing bootstrap support with five of the six genes used in this study, excluding ITS2.

S1 Table. Taxon sampling of Scarabaeidae by genus and species, including collection locality and specimen voucher number.

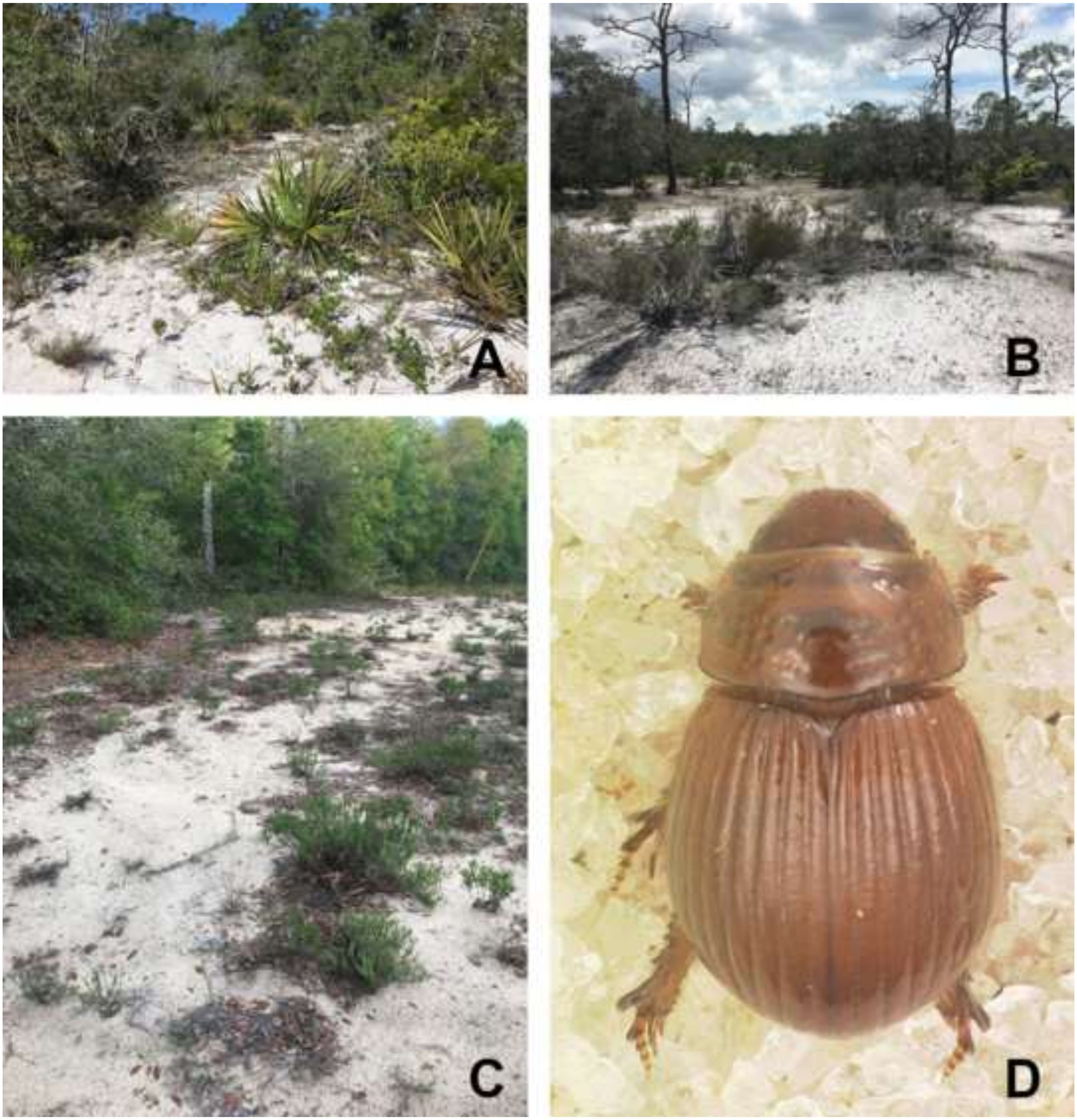
A
B
C
D

Figure 2

Click here to access/download;Figure;Fig2.tif

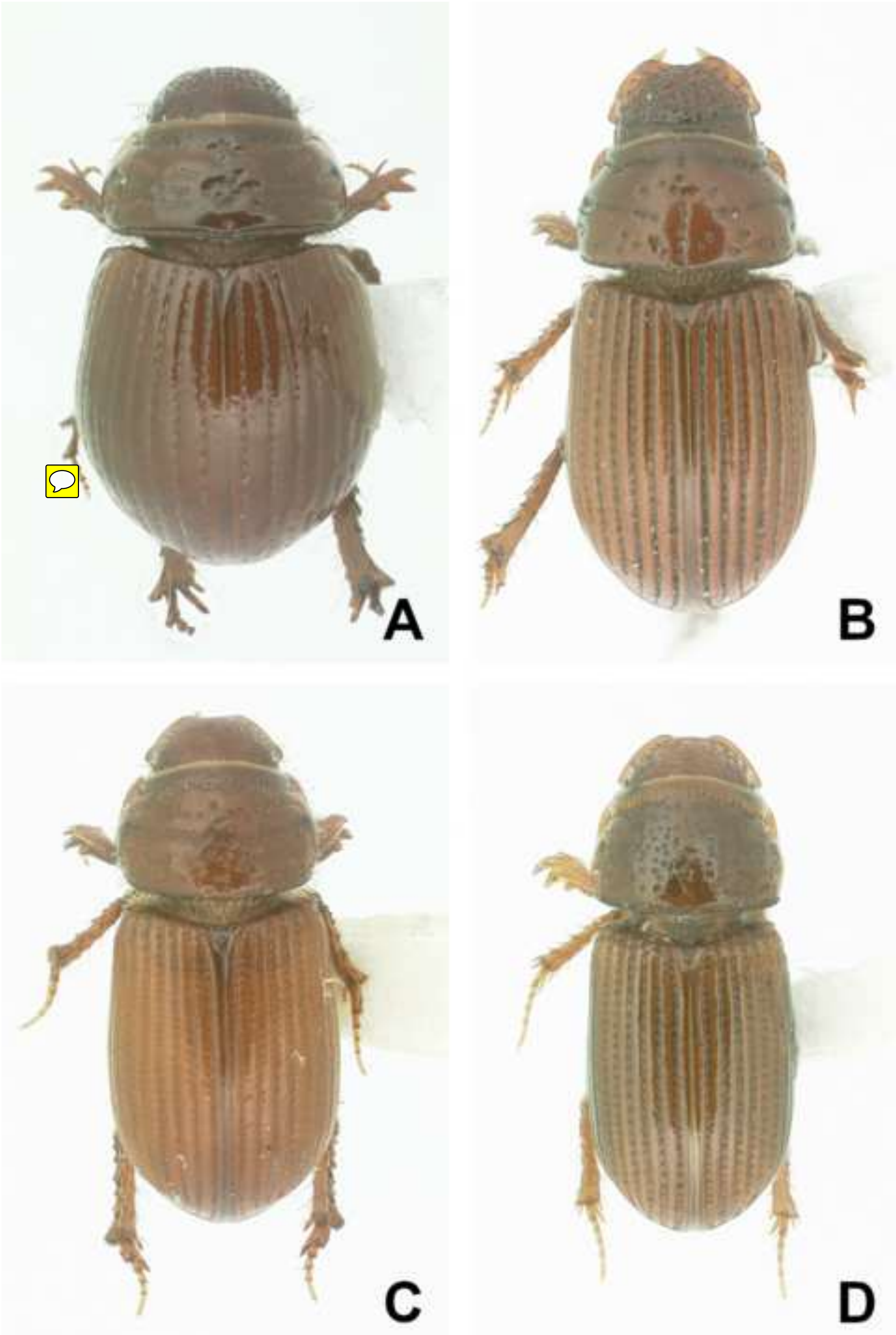

Figure 3

Click here to access/download;Figure;Fig3.tif

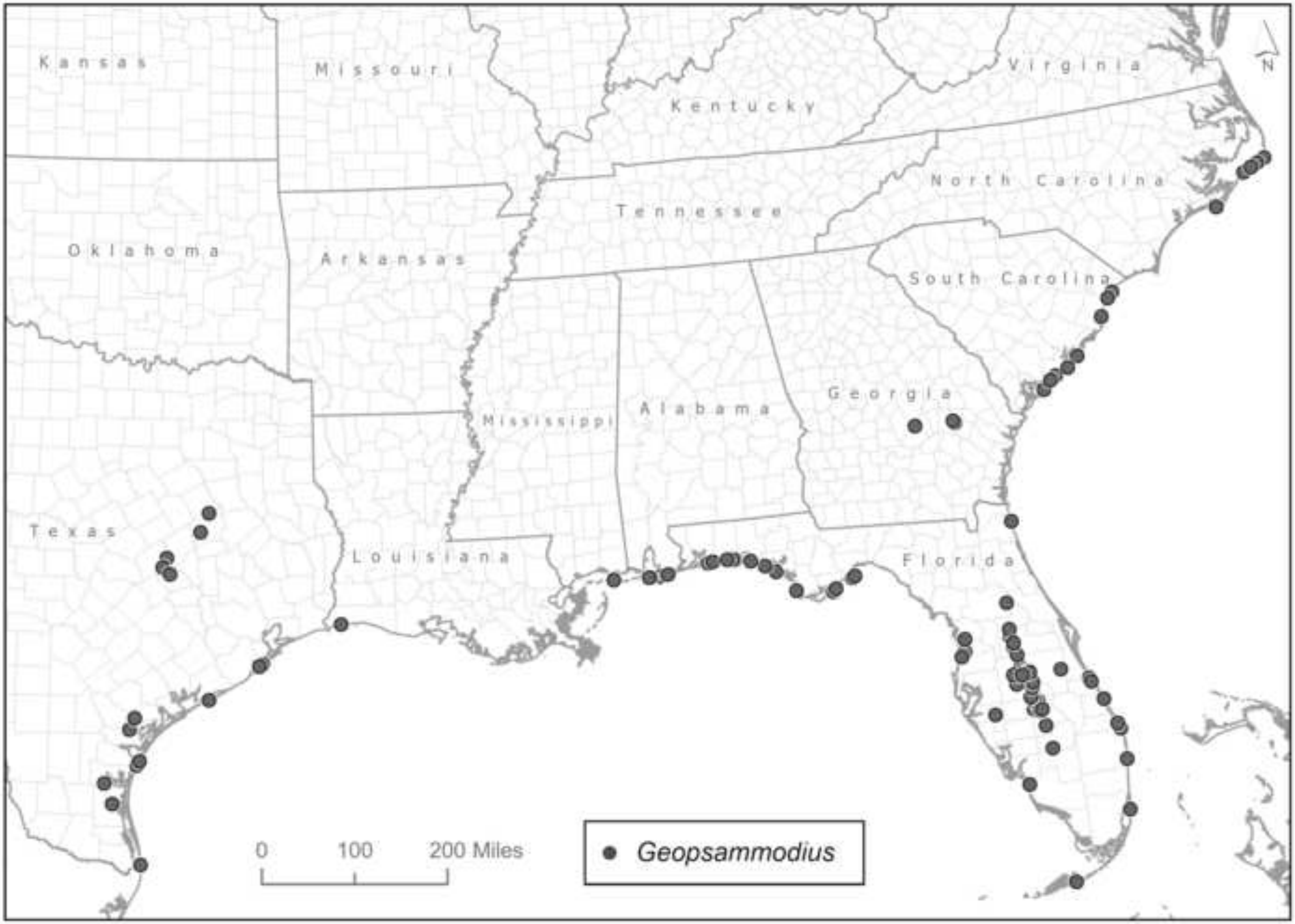

Figure 4

Click here to access/download;Figure;Fig4.tif

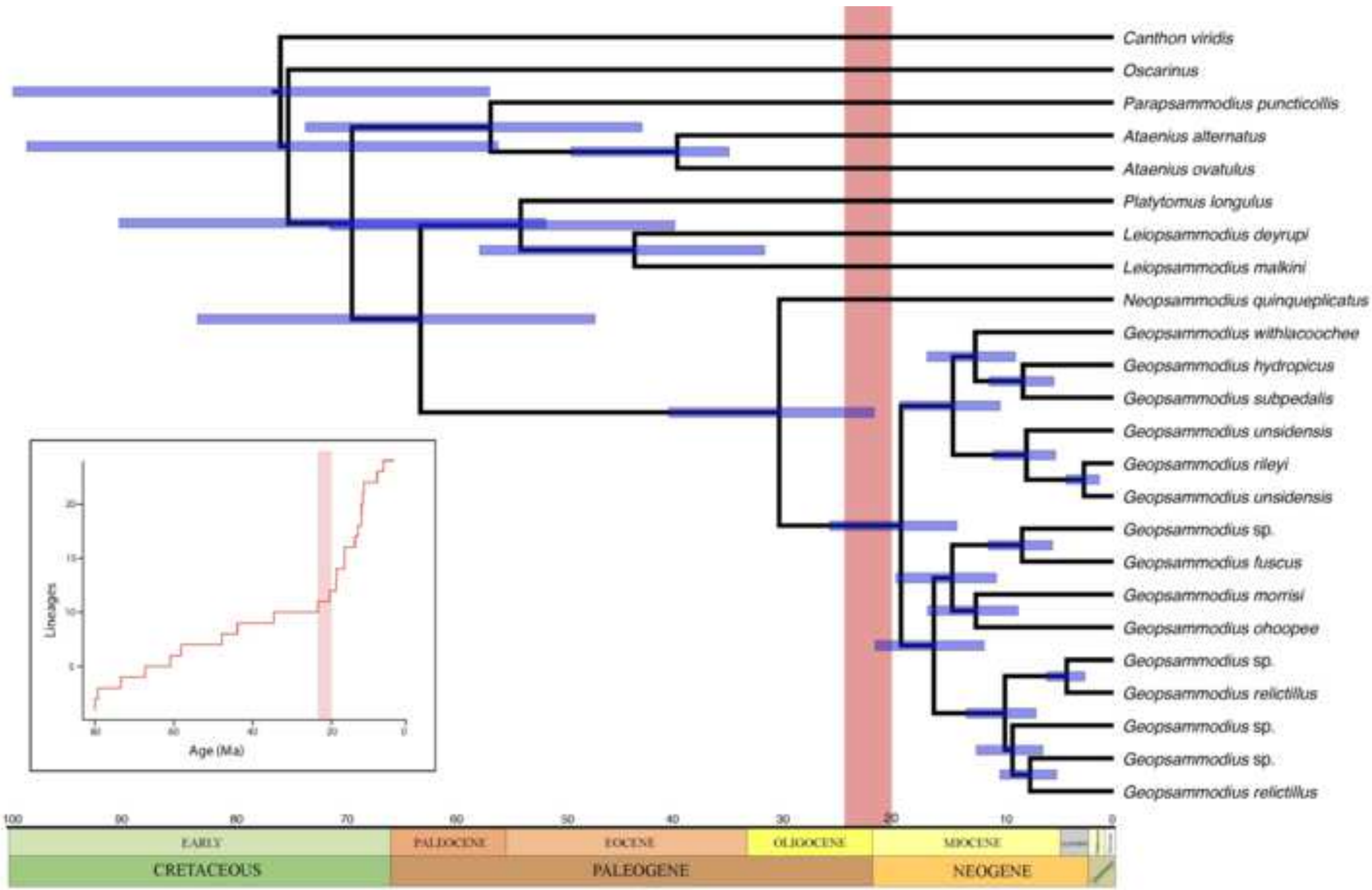

Figure 5

Click here to access/download;Figure;Fig5.tif

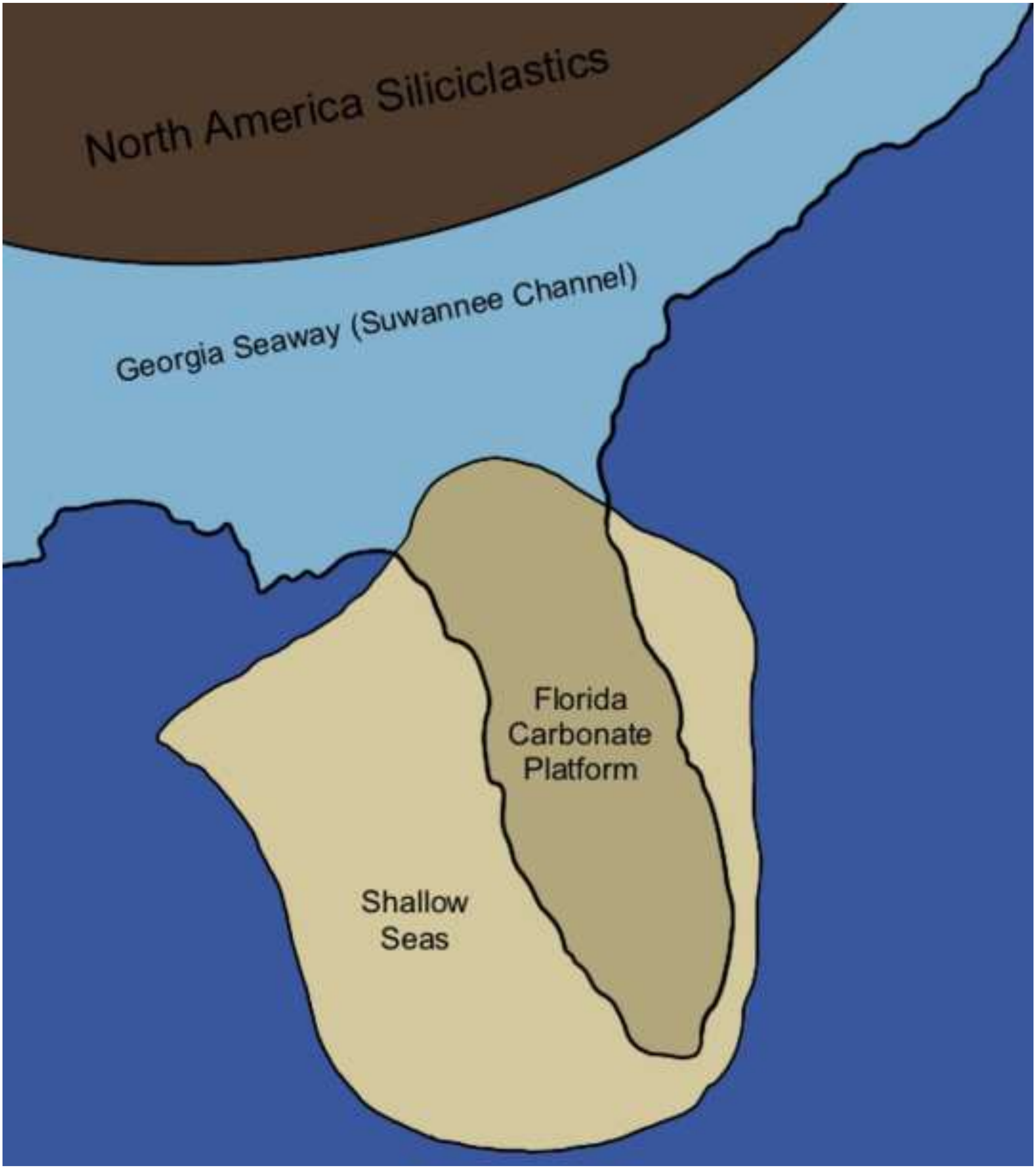

Supporting Information S1_Fig

Click here to access/download
Supporting Information
S1_Fig.tif

Click here to access/download
Supporting Information
S2_Fig.tif

Click here to access/download
Supporting Information
S1_Table.xlsx
