# Supplementary material for: Phylogeography of Geopsammodius Gordon and Pittino, 1992 (Coleoptera: Scarabaeidae: Aphodiinae): A radiation of endemic psammophilic beetles
Source: PLoS One. 2026 Aug 12;21(8):e0351948. doi: 10.1371/journal.pone.0351948 (PMC13466012; doi:10.1371/journal.pone.0351948)
Supplement: Supplementary file 3 [file pone.0351948.s007.docx]

**Response to Reviewers**

**Author’s response is in blue below**

Dear Editor,

Overall, the study addresses an interesting topic, but several aspects require revision in revised manuscript. I have outlined the following suggestions below.

I.The author name and year of publication for a scientific name (genus or species) should be included the first time it appears in the manuscript. In subsequent mentions, the author name and year may be omitted.

Authors and years have been added to the necessary taxa. We removed a couple instances where it was written on subsequent appearances of the name.

II.Several sentences lack the necessary references and require careful revision to include appropriate citations.

We have added references to those places indicated in the text.

III.The name of the museum where the samples were examined should be provided, along with details regarding specimen deposition. Additionally, the reference(s) used for taxonomic identification should be cited.

The museums where the specimens are deposited are listed in the methods. We added a sentence specifying the use of Skelley, 2006 for identification.

IV.In the phylogenetic trees, species names should be accompanied by their corresponding codes, as several species are listed under "spp." This will help distinguish them clearly and facilitate future studies.

We have updated the figure so all species names are now corresponding to their extraction codes.

V.A scale bar should be added to the trees, and the tribe or genus should be indicated using labels placed directly on the figures.

We have updated the figure so that the tribes are labeled along the side. Note that the scale bar is located at the bottom of the figure in the main text, as well as in the supplementary figures.

VI.Several references are missing page numbers and should be updated accordingly.

Page numbers have been updated in the text.

VII.The quality of the photographs could be improved using image editing software (e.g., Photoshop) by adjusting brightness and contrast.

The images have had the contrast increased for easier viewing. For final publication we can submit higher-resolution images. The photos were compressed when uploaded to the submission portal.

VIII.Figure 3 currently displays only dots without indicating any species. It would be better to color the dots to represent different species, or to label them with numbers and provide a corresponding species list.

Species are very close geographically and many colored dots would overlap, rendering the colors useless. The intent of this figure is to convey the distribution of the genus in the southeastern United States. There would need to be four separate maps at very fine scale to realistically represent the individual species. I agree this would be warranted and useful in the context of a generic revision but not for this study.

IX. While the study uses six genes to construct the phylogeny, it doesn’t mention how many species or individuals from the genus Geopsammodius were sampled. A larger and more representative sample of species within the genus and the tribe Psammodiini would help to strengthen the conclusions drawn about species relationships and divergence. Without comprehensive sampling, the results may not be fully representative of the diversity within the genus or tribe.

We edited the sentence about taxon sampling to clarify that we had 15 specimens representing 9 of the 11 species. It is true that including more taxa is always better. We have places in the discussion addressing those few species we were not able to include.

X. The estimation of divergence times relies heavily on molecular clocks, which can be prone to error if the molecular clock is not calibrated properly. This can lead to inaccurate age estimates, especially if the underlying assumptions (such as constant mutation rates) do not hold true across all species. The study doesn’t clarify how the molecular clock was calibrated or how much uncertainty there might be in the divergence time estimates.

The molecular clock used in our methods was based on that of insect mitochondrial DNA. This is a standard clock rate used throughout the literature, which we only applied to the mtDNA portion of our dataset. We recognize the uncertainty in any divergence time estimation and have added to the text the 95% HPD where appropriate to signify this uncertainty.

XI. The finding that Parapsammodius is placed outside Psammodiini indicates the need for future taxonomic revision. However, this raises questions about the current taxonomic boundaries and classifications. The lack of detailed discussion on why Parapsammodius was previously thought to belong within Psammodiini and the potential implications for other related genera leaves some gaps in understanding the broader taxonomic landscape.

Please refer to the first several paragraphs of the discussion (systematics) which discuss the morphological characters that have made distinguishing Eupariini and Psammodini so difficult. As the reviewer mentioned before, our taxon sampling is limited so we avoid making too many conclusions regarding the higher-level classification of these species.

Reviewer #1: Following needs to be clarified in the relevant sections:

1. While the study uses six genes to construct the phylogeny, it doesn’t mention how many species or individuals from the genus Geopsammodius were sampled. A larger and more representative sample of species within the genus and the tribe Psammodiini would help to strengthen the conclusions drawn about species relationships and divergence. Without comprehensive sampling, the results may not be fully representative of the diversity within the genus or tribe.

We edited the sentence about taxon sampling to clarify that we had 15 specimens representing 9 of the 11 species. We do not have a broader taxon sampling of this family so avoid discussion tribal-level classification in detail. Our focus for this paper was on the genus *Geopsammodius* specifically.

2. The estimation of divergence times relies heavily on molecular clocks, which can be prone to error if the molecular clock is not calibrated properly. This can lead to inaccurate age estimates, especially if the underlying assumptions (such as constant mutation rates) do not hold true across all species. The study doesn’t clarify how the molecular clock was calibrated or how much uncertainty there might be in the divergence time estimates.

The molecular clock used in our methods was based on that of insect mitochondrial DNA. This is a standard clock rate used throughout the literature, which we only applied to the mtDNA portion of our dataset. We recognize the uncertainty in any divergence time estimation and have added to the text the 95% HPD where appropriate to signify this uncertainty.

3. The finding that Parapsammodius is placed outside Psammodiini indicates the need for future taxonomic revision. However, this raises questions about the current taxonomic boundaries and classifications. The lack of detailed discussion on why Parapsammodius was previously thought to belong within Psammodiini and the potential implications for other related genera leaves some gaps in understanding the broader taxonomic landscape.

Please refer to the first several paragraphs of the discussion (systematics) which discuss the morphological characters that have made distinguishing Eupariini and Psammodini so difficult. As the reviewer mentioned before, our taxon sampling is limited so we avoid making too many conclusions regarding the higher-level classification of these species.

Reviewer #2: The manuscript may be accepted for publication after addressing minor comments which i marked directly in the file. There are some minor corrections such such references are missing and author and years are not given for generic names.

We have addressed all the comments directly in the manuscript.

**Specific comments in the text:**

Line 27 & 33: Author and year added to text

Line 47: updated

Line 69: We have updated the sentence to reflect new taxonomic names

Line 87: We retained the sentence about common names as the point of the sentence is to introduce what Aphodiinae are commonly known for. The type of dung is not relevant here, and we recognize common names are not always scientifically accurate, as we explain in the following sentences.

Line 91: References added.

Line 96: References added.

Line 114: References added

Line 116: updated

Line 161: Museums where voucher specimens are deposited is listed below.

Line 164: added sentence about using Skelley 2006 for identifications

Line 279: Updated text

Line 275: Not the first place the genus is mentioned. See line 240.

Line 195-196: References added.
