# Supplementary material for: Phylogeography of Geopsammodius Gordon and Pittino, 1992 (Coleoptera: Scarabaeidae: Aphodiinae): A radiation of endemic psammophilic beetles
Source: PLoS One. 2026 Aug 12;21(8):e0351948. doi: 10.1371/journal.pone.0351948 (PMC13466012; doi:10.1371/journal.pone.0351948)
Supplement: Peer review history

# Peer review: Decision Letter 0

**DOI:** 10.1371/journal.pone.0351948.r001

**Authors:** Muhammad Asghar Hassan

1 Apr 2026

Dear Dr. Schnepp,

Thank you for submitting your manuscript to PLOS ONE. After careful consideration, we feel that it has merit but does not fully meet PLOS ONE’s publication criteria as it currently stands. Therefore, we invite you to submit a revised version of the manuscript that addresses the points raised during the review process.

Please submit your revised manuscript by May 16 2026 11:59PM. If you will need more time than this to complete your revisions, please reply to this message or contact the journal office at plosone@plos.org. When you're ready to submit your revision, log on to https://www.editorialmanager.com/pone/ and select the 'Submissions Needing Revision' folder to locate your manuscript file.

- A letter that responds to each point raised by the academic editor and reviewer(s). You should upload this letter as a separate file labeled 'Response to Reviewers'.
- A marked-up copy of your manuscript that highlights changes made to the original version. You should upload this as a separate file labeled 'Revised Manuscript with Track Changes'.
- An unmarked version of your revised paper without tracked changes. You should upload this as a separate file labeled 'Manuscript'.

If you would like to make changes to your financial disclosure, please include your updated statement in your cover letter. Guidelines for resubmitting your figure files are available below the reviewer comments at the end of this letter.

If applicable, we recommend that you deposit your laboratory protocols in protocols.io to enhance the reproducibility of your results. Protocols.io assigns your protocol its own identifier (DOI) so that it can be cited independently in the future. For instructions see: https://journals.plos.org/plosone/s/submission-guidelines#loc-laboratory-protocols. Additionally, PLOS ONE offers an option for publishing peer-reviewed Lab Protocol articles, which describe protocols hosted on protocols.io. Read more information on sharing protocols at https://plos.org/protocols?utm_medium=editorial-email&utm_source=authorletters&utm_campaign=protocols.

As the corresponding author, your ORCID iD is verified in the submission system and will appear in the published article. PLOS supports the use of ORCID, and we encourage all coauthors to register for an ORCID iD and use it as well. Please encourage your coauthors to verify their ORCID iD within the submission system before final acceptance, as unverified ORCID iDs will not appear in the published article. *Only*  the individual author can complete the verification step; PLOS staff *cannot*  verify ORCID iDs on behalf of authors.

We look forward to receiving your revised manuscript.

Kind regards,

Muhammad Asghar Hassan, Ph.D

Academic Editor

PLOS One

Journal Requirements:

When submitting your revision, we need you to address these additional requirements.

1. Please ensure that your manuscript meets PLOS ONE's style requirements, including those for file naming. The PLOS ONE style templates can be found at

https://journals.plos.org/plosone/s/file?id=wjVg/PLOSOne_formatting_sample_main_body.pdf and https://journals.plos.org/plosone/s/file?id=ba62/PLOSOne_formatting_sample_title_authors_affiliations.pdf

2. In your Methods section, please provide additional information regarding the permits you obtained for the work. Please ensure you have included the full name of the authority that approved the field site access and, if no permits were required, a brief statement explaining why.

3. When completing the data availability statement of the submission form, you indicated that you will make your data available on acceptance. We strongly recommend all authors decide on a data sharing plan before acceptance, as the process can be lengthy and hold up publication timelines. Please note that, though access restrictions are acceptable now, your entire data will need to be made freely accessible if your manuscript is accepted for publication. This policy applies to all data except where public deposition would breach compliance with the protocol approved by your research ethics board. If you are unable to adhere to our open data policy, please kindly revise your statement to explain your reasoning and we will seek the editor's input on an exemption. Please be assured that, once you have provided your new statement, the assessment of your exemption will not hold up the peer review process.

4. We note that Figures 1 and 2 in your submission contain images which may be copyrighted. All PLOS content is published under the Creative Commons Attribution License (CC BY 4.0), which means that the manuscript, images, and Supporting Information files will be freely available online, and any third party is permitted to access, download, copy, distribute, and use these materials in any way, even commercially, with proper attribution. For more information, see our copyright guidelines: http://journals.plos.org/plosone/s/licenses-and-copyright.

We require you to either (1) present written permission from the copyright holder to publish these figures specifically under the CC BY 4.0 license, or (2) remove the figures from your submission:

1. You may seek permission from the original copyright holder of Figures 1 and 2 to publish the content specifically under the CC BY 4.0 license.

We recommend that you contact the original copyright holder with the Content Permission Form (http://journals.plos.org/plosone/s/file?id=7c09/content-permission-form.pdf) and the following text:

“I request permission for the open-access journal PLOS ONE to publish XXX under the Creative Commons Attribution License (CCAL) CC BY 4.0 (http://creativecommons.org/licenses/by/4.0/). Please be aware that this license allows unrestricted use and distribution, even commercially, by third parties. Please reply and provide explicit written permission to publish XXX under a CC BY license and complete the attached form.”

Please upload the completed Content Permission Form or other proof of granted permissions as an "Other" file with your submission.

In the figure caption of the copyrighted figure, please include the following text: “Reprinted from [ref] under a CC BY license, with permission from [name of publisher], original copyright [original copyright year].”

2. If you are unable to obtain permission from the original copyright holder to publish these figures under the CC BY 4.0 license or if the copyright holder’s requirements are incompatible with the CC BY 4.0 license, please either i) remove the figure or ii) supply a replacement figure that complies with the CC BY 4.0 license. Please check copyright information on all replacement figures and update the figure caption with source information. If applicable, please specify in the figure caption text when a figure is similar but not identical to the original image and is therefore for illustrative purposes only.

5. We note that Figures 3 and 5 in your submission contain map images which may be copyrighted. All PLOS content is published under the Creative Commons Attribution License (CC BY 4.0), which means that the manuscript, images, and Supporting Information files will be freely available online, and any third party is permitted to access, download, copy, distribute, and use these materials in any way, even commercially, with proper attribution. For these reasons, we cannot publish previously copyrighted maps or satellite images created using proprietary data, such as Google software (Google Maps, Street View, and Earth). For more information, see our copyright guidelines: http://journals.plos.org/plosone/s/licenses-and-copyright.

We require you to either (1) present written permission from the copyright holder to publish these figures specifically under the CC BY 4.0 license, or (2) remove the figures from your submission:

1. You may seek permission from the original copyright holder of Figures 3 and 5 to publish the content specifically under the CC BY 4.0 license.

We recommend that you contact the original copyright holder with the Content Permission Form (http://journals.plos.org/plosone/s/file?id=7c09/content-permission-form.pdf) and the following text:

“I request permission for the open-access journal PLOS ONE to publish XXX under the Creative Commons Attribution License (CCAL) CC BY 4.0 (http://creativecommons.org/licenses/by/4.0/). Please be aware that this license allows unrestricted use and distribution, even commercially, by third parties. Please reply and provide explicit written permission to publish XXX under a CC BY license and complete the attached form.”

Please upload the completed Content Permission Form or other proof of granted permissions as an "Other" file with your submission.

In the figure caption of the copyrighted figure, please include the following text: “Reprinted from [ref] under a CC BY license, with permission from [name of publisher], original copyright [original copyright year].”

2. If you are unable to obtain permission from the original copyright holder to publish these figures under the CC BY 4.0 license or if the copyright holder’s requirements are incompatible with the CC BY 4.0 license, please either i) remove the figure or ii) supply a replacement figure that complies with the CC BY 4.0 license. Please check copyright information on all replacement figures and update the figure caption with source information. If applicable, please specify in the figure caption text when a figure is similar but not identical to the original image and is therefore for illustrative purposes only.

The following resources for replacing copyrighted map figures may be helpful:

USGS National Map Viewer (public domain): http://viewer.nationalmap.gov/viewer/

The Gateway to Astronaut Photography of Earth (public domain): http://eol.jsc.nasa.gov/sseop/clickmap/

Maps at the CIA (public domain): https://www.cia.gov/library/publications/the-world-factbook/index.html and https://www.cia.gov/library/publications/cia-maps-publications/index.html

NASA Earth Observatory (public domain): http://earthobservatory.nasa.gov/

Landsat: http://landsat.visibleearth.nasa.gov/

USGS EROS (Earth Resources Observatory and Science (EROS) Center) (public domain): http://eros.usgs.gov/#

Natural Earth (public domain): http://www.naturalearthdata.com/

If the reviewer comments include a recommendation to cite specific previously published works, please review and evaluate these publications to determine whether they are relevant and should be cited. There is no requirement to cite these works unless the editor has indicated otherwise.

Please review your reference list to ensure that it is complete and correct. If you have cited papers that have been retracted, please include the rationale for doing so in the manuscript text, or remove these references and replace them with relevant current references. Any changes to the reference list should be mentioned in the rebuttal letter that accompanies your revised manuscript. If you need to cite a retracted article, indicate the article’s retracted status in the References list and also include a citation and full reference for the retraction notice.

Additional Editor Comments:

Dear Editor,

Overall, the study addresses an interesting topic, but several aspects require revision in revised manuscript. I have outlined the following suggestions below.

I.The author name and year of publication for a scientific name (genus or species) should be included the first time it appears in the manuscript. In subsequent mentions, the author name and year may be omitted.

II.Several sentences lack the necessary references and require careful revision to include appropriate citations.

III.The name of the museum where the samples were examined should be provided, along with details regarding specimen deposition. Additionally, the reference(s) used for taxonomic identification should be cited.

IV.In the phylogenetic trees, species names should be accompanied by their corresponding codes, as several species are listed under "spp." This will help distinguish them clearly and facilitate future studies.

V.A scale bar should be added to the trees, and the tribe or genus should be indicated using labels placed directly on the figures.

VI.Several references are missing page numbers and should be updated accordingly.

VII.The quality of the photographs could be improved using image editing software (e.g., Photoshop) by adjusting brightness and contrast.

VIII.Figure 3 currently displays only dots without indicating any species. It would be better to color the dots to represent different species, or to label them with numbers and provide a corresponding species list.

IX. While the study uses six genes to construct the phylogeny, it doesn’t mention how many species or individuals from the genus Geopsammodius were sampled. A larger and more representative sample of species within the genus and the tribe Psammodiini would help to strengthen the conclusions drawn about species relationships and divergence. Without comprehensive sampling, the results may not be fully representative of the diversity within the genus or tribe.

X. The estimation of divergence times relies heavily on molecular clocks, which can be prone to error if the molecular clock is not calibrated properly. This can lead to inaccurate age estimates, especially if the underlying assumptions (such as constant mutation rates) do not hold true across all species. The study doesn’t clarify how the molecular clock was calibrated or how much uncertainty there might be in the divergence time estimates.

XI. The finding that Parapsammodius is placed outside Psammodiini indicates the need for future taxonomic revision. However, this raises questions about the current taxonomic boundaries and classifications. The lack of detailed discussion on why Parapsammodius was previously thought to belong within Psammodiini and the potential implications for other related genera leaves some gaps in understanding the broader taxonomic landscape.

[Note: HTML markup is below. Please do not edit.]

Reviewers' comments:

Reviewer's Responses to Questions

**Comments to the Author**

1. Is the manuscript technically sound, and do the data support the conclusions?

Reviewer #1: Yes

Reviewer #2: Yes

**********

2. Has the statistical analysis been performed appropriately and rigorously?

Reviewer #1: N/A

Reviewer #2: Yes

**********

3. Have the authors made all data underlying the findings in their manuscript fully available?

The PLOS Data policy

Reviewer #1: Yes

Reviewer #2: Yes

**********

4. Is the manuscript presented in an intelligible fashion and written in standard English?

Reviewer #1: Yes

Reviewer #2: Yes

**********

Reviewer #1: Following needs to be clarified in the relevant sections:

1. While the study uses six genes to construct the phylogeny, it doesn’t mention how many species or individuals from the genus Geopsammodius were sampled. A larger and more representative sample of species within the genus and the tribe Psammodiini would help to strengthen the conclusions drawn about species relationships and divergence. Without comprehensive sampling, the results may not be fully representative of the diversity within the genus or tribe.

2. The estimation of divergence times relies heavily on molecular clocks, which can be prone to error if the molecular clock is not calibrated properly. This can lead to inaccurate age estimates, especially if the underlying assumptions (such as constant mutation rates) do not hold true across all species. The study doesn’t clarify how the molecular clock was calibrated or how much uncertainty there might be in the divergence time estimates.

3. The finding that Parapsammodius is placed outside Psammodiini indicates the need for future taxonomic revision. However, this raises questions about the current taxonomic boundaries and classifications. The lack of detailed discussion on why Parapsammodius was previously thought to belong within Psammodiini and the potential implications for other related genera leaves some gaps in understanding the broader taxonomic landscape.

Reviewer #2: The manuscript may be accepted for publication after addressing minor comments which i marked directly in the file. There are some minor corrections such such references are missing and author and years are not given for generic names.

**********

what does this mean?). If published, this will include your full peer review and any attached files.

If you choose “no”, your identity will remain anonymous but your review may still be made public.

**Do you want your identity to be public for this peer review?**  For information about this choice, including consent withdrawal, please see our Privacy Policy

Reviewer #1: No

Reviewer #2: **Yes:** Dr Devanshu Gupta

**********

[NOTE: If reviewer comments were submitted as an attachment file, they will be attached to this email and accessible via the submission site. Please log into your account, locate the manuscript record, and check for the action link "View Attachments". If this link does not appear, there are no attachment files.]

To ensure your figures meet our technical requirements, please review our figure guidelines: https://journals.plos.org/plosone/s/figures

You may also use PLOS’s free figure tool, NAAS, to help you prepare publication quality figures: https://journals.plos.org/plosone/s/figures#loc-tools-for-figure-preparation.

NAAS will assess whether your figures meet our technical requirements by comparing each figure against our figure specifications.

Attachment Submitted filename: PONE-D-26-10786.pdf

Attachment Submitted filename: PONE-D-26-10786(R).pdf

# Author comment: Author response to Decision Letter 1

**DOI:** 10.1371/journal.pone.0351948.r002

23 May 2026

Responses to author comments are under each question below and are preceded by "--".

I.The author name and year of publication for a scientific name (genus or species) should be included the first time it appears in the manuscript. In subsequent mentions, the author name and year may be omitted.

----Authors and years have been added to the necessary taxa. We removed a couple instances where it was written on subsequent appearances of the name.

II.Several sentences lack the necessary references and require careful revision to include appropriate citations.

----We have added references to those places indicated in the text.

III.The name of the museum where the samples were examined should be provided, along with details regarding specimen deposition. Additionally, the reference(s) used for taxonomic identification should be cited.

----The museums where the specimens are deposited are listed in the methods. We added a sentence specifying the use of Skelley, 2006 for identification.

IV.In the phylogenetic trees, species names should be accompanied by their corresponding codes, as several species are listed under "spp." This will help distinguish them clearly and facilitate future studies.

----We have updated the figure so all species names are now corresponding to their extraction codes.

V.A scale bar should be added to the trees, and the tribe or genus should be indicated using labels placed directly on the figures.

----We have updated the figure so that the tribes are labeled along the side. Note that the scale bar is located at the bottom of the figure in the main text, as well as in the supplementary figures.

VI.Several references are missing page numbers and should be updated accordingly.

----Page numbers have been updated in the text.

VII.The quality of the photographs could be improved using image editing software (e.g., Photoshop) by adjusting brightness and contrast.

----The images have had the contrast increased for easier viewing. For final publication we can submit higher-resolution images. The photos were compressed when uploaded to the submission portal.

VIII.Figure 3 currently displays only dots without indicating any species. It would be better to color the dots to represent different species, or to label them with numbers and provide a corresponding species list.

----Species are very close geographically and many colored dots would overlap, rendering the colors useless. The intent of this figure is to convey the distribution of the genus in the southeastern United States. There would need to be four separate maps at very fine scale to realistically represent the individual species. I agree this would be warranted and useful in the context of a generic revision but not for this study.

IX. While the study uses six genes to construct the phylogeny, it doesn’t mention how many species or individuals from the genus Geopsammodius were sampled. A larger and more representative sample of species within the genus and the tribe Psammodiini would help to strengthen the conclusions drawn about species relationships and divergence. Without comprehensive sampling, the results may not be fully representative of the diversity within the genus or tribe.

----We edited the sentence about taxon sampling to clarify that we had 15 specimens representing 9 of the 11 species. It is true that including more taxa is always better. We have places in the discussion addressing those few species we were not able to include.

X. The estimation of divergence times relies heavily on molecular clocks, which can be prone to error if the molecular clock is not calibrated properly. This can lead to inaccurate age estimates, especially if the underlying assumptions (such as constant mutation rates) do not hold true across all species. The study doesn’t clarify how the molecular clock was calibrated or how much uncertainty there might be in the divergence time estimates.

----The molecular clock used in our methods was based on that of insect mitochondrial DNA. This is a standard clock rate used throughout the literature, which we only applied to the mtDNA portion of our dataset. We recognize the uncertainty in any divergence time estimation and have added to the text the 95% HPD where appropriate to signify this uncertainty.

XI. The finding that Parapsammodius is placed outside Psammodiini indicates the need for future taxonomic revision. However, this raises questions about the current taxonomic boundaries and classifications. The lack of detailed discussion on why Parapsammodius was previously thought to belong within Psammodiini and the potential implications for other related genera leaves some gaps in understanding the broader taxonomic landscape.

----Please refer to the first several paragraphs of the discussion (systematics) which discuss the morphological characters that have made distinguishing Eupariini and Psammodini so difficult. As the reviewer mentioned before, our taxon sampling is limited so we avoid making too many conclusions regarding the higher-level classification of these species.

Reviewer #1: Following needs to be clarified in the relevant sections:

1. While the study uses six genes to construct the phylogeny, it doesn’t mention how many species or individuals from the genus Geopsammodius were sampled. A larger and more representative sample of species within the genus and the tribe Psammodiini would help to strengthen the conclusions drawn about species relationships and divergence. Without comprehensive sampling, the results may not be fully representative of the diversity within the genus or tribe.

----We edited the sentence about taxon sampling to clarify that we had 15 specimens representing 9 of the 11 species. We do not have a broader taxon sampling of this family so avoid discussion tribal-level classification in detail. Our focus for this paper was on the genus Geopsammodius specifically.

2. The estimation of divergence times relies heavily on molecular clocks, which can be prone to error if the molecular clock is not calibrated properly. This can lead to inaccurate age estimates, especially if the underlying assumptions (such as constant mutation rates) do not hold true across all species. The study doesn’t clarify how the molecular clock was calibrated or how much uncertainty there might be in the divergence time estimates.

----The molecular clock used in our methods was based on that of insect mitochondrial DNA. This is a standard clock rate used throughout the literature, which we only applied to the mtDNA portion of our dataset. We recognize the uncertainty in any divergence time estimation and have added to the text the 95% HPD where appropriate to signify this uncertainty.

3. The finding that Parapsammodius is placed outside Psammodiini indicates the need for future taxonomic revision. However, this raises questions about the current taxonomic boundaries and classifications. The lack of detailed discussion on why Parapsammodius was previously thought to belong within Psammodiini and the potential implications for other related genera leaves some gaps in understanding the broader taxonomic landscape.

----Please refer to the first several paragraphs of the discussion (systematics) which discuss the morphological characters that have made distinguishing Eupariini and Psammodini so difficult. As the reviewer mentioned before, our taxon sampling is limited so we avoid making too many conclusions regarding the higher-level classification of these species.

Reviewer #2: The manuscript may be accepted for publication after addressing minor comments which i marked directly in the file. There are some minor corrections such such references are missing and author and years are not given for generic names.

----We have addressed all the comments directly in the manuscript.

Specific comments in the text:

----Line 27 & 33: Author and year added to text

----Line 47: updated

----Line 69: We have updated the sentence to reflect new taxonomic names

----Line 87: We retained the sentence about common names as the point of the sentence is to introduce what Aphodiinae are commonly known for. The type of dung is not relevant here, and we recognize common names are not always scientifically accurate, as we explain in the following sentences.

----Line 91: References added.

----Line 96: References added.

----Line 114: References added

----Line 116: updated

----Line 161: Museums where voucher specimens are deposited is listed below.

----Line 164: added sentence about using Skelley 2006 for identifications

----Line 279: Updated text

----Line 275: Not the first place the genus is mentioned. See line 240.

----Line 195-196: References added.

Attachment Submitted filename: Response to Reviewers.docx

# Editor report: Decision Letter 1

**DOI:** 10.1371/journal.pone.0351948.r003

**Authors:** Muhammad Asghar Hassan

3 Jun 2026

Phylogeography of Geopsammodius Gordon and Pittino, 1992 (Coleoptera: Scarabaeidae: Aphodiinae): a radiation of endemic psammophilic beetles

PONE-D-26-10786R1

Dear Dr. Kyle E. Schnepp,

We’re pleased to inform you that your manuscript has been judged scientifically suitable for publication and will be formally accepted for publication once it meets all outstanding technical requirements.

Within one week, you’ll receive an e-mail detailing the required amendments. When these have been addressed, you’ll receive a formal acceptance letter and your manuscript will be scheduled for publication.

An invoice will be generated when your article is formally accepted. Please note, if your institution has a publishing partnership with PLOS and your article meets the relevant criteria, all or part of your publication costs will be covered. Please make sure your user information is up-to-date by logging into Editorial Manager at Editorial Manager® and clicking the ‘Update My Information' link at the top of the page. For questions related to billing, please contact billing support.

If your institution or institutions have a press office, please notify them about your upcoming paper to help maximize its impact. If they’ll be preparing press materials, please inform our press team as soon as possible -- no later than 48 hours after receiving the formal acceptance. Your manuscript will remain under strict press embargo until 2 pm Eastern Time on the date of publication. For more information, please contact onepress@plos.org.

Kind regards,

Muhammad Asghar Hassan, Ph.D

Academic Editor

PLOS One

Additional Editor Comments (optional):

Dear Editor,

The authors have adequately addressed most of the comments and incorporated the required corrections. Therefore, after the following minor revisions are addressed, I recommend this manuscript for acceptance and publication.

Minor Comments

1.Line 75 – Please add the relevant reference.

2.Line 86 – MJ Paulsen, personal communication

1.The authors should provide evidence of this personal communication (e.g., email correspondence or written documentation).

2.If Dr. Paulsen agrees, he should also be acknowledged appropriately in the Acknowledgements section.

3.Lines 93–95 – Please provide the relevant reference(s).

4.Lines 108–112 – Please add relevant and updated references supporting the diagnosis of this tribe.

5.Line 120 – Revise to: the same tribe within Aphodiinae.

6.Lines 122–125 – Supporting reference(s) are required.

7.Lines 131–132 – The genus name appearing before -group should be italicized.

8.Lines 133–136 – The genus name Geopsammodius is repeated several times. Please retain the full genus name only at its first mention and abbreviate it thereafter.

9.Line 200 – Add sp. after Aphodius.

10.Line 427 – The authors acknowledge several colleagues. Please confirm that these individuals are aware of and have consented to being acknowledged in the manuscript.

Based on the revised manuscript and subject to the satisfactory correction of the above minor issues, I recommend acceptance for publication. Please also provide high quality figures.

# Editor report: Acceptance letter

**DOI:** 10.1371/journal.pone.0351948.r004

**Authors:** Muhammad Asghar Hassan

PONE-D-26-10786R1

PLOS One

Dear Dr. Schnepp,

I'm pleased to inform you that your manuscript has been deemed suitable for publication in PLOS One. Congratulations! Your manuscript is now being handed over to our production team.

At this stage, our production department will prepare your paper for publication. This includes ensuring the following:

* All references, tables, and figures are properly cited

* All relevant supporting information is included in the manuscript submission,

* There are no issues that prevent the paper from being properly typeset

You will receive further instructions from the production team, including instructions on how to review your proof when it is ready. Please keep in mind that we are working through a large volume of accepted articles, so please give us a few days to review your paper and let you know the next and final steps.

Lastly, if your institution or institutions have a press office, please let them know about your upcoming paper now to help maximize its impact. If they'll be preparing press materials, please inform our press team within the next 48 hours. Your manuscript will remain under strict press embargo until 2 pm Eastern Time on the date of publication. For more information, please contact onepress@plos.org.

You will receive an invoice from PLOS for your publication fee after your manuscript has reached the completed accept phase. If you receive an email requesting payment before acceptance or for any other service, this may be a phishing scheme. Learn how to identify phishing emails and protect your accounts at https://explore.plos.org/phishing.

If we can help with anything else, please email us at customercare@plos.org.

Thank you for submitting your work to PLOS ONE and supporting open access.

Kind regards,

PLOS ONE Editorial Office Staff

on behalf of

Dr. Muhammad Asghar Hassan

Academic Editor

PLOS One
